# Nanoarchitectonics and Simulation on the Molecular-Level Interactions between *p-*Sulfonic Acid Calix[4]arene and Langmuir Monolayers Representing Healthy and Cancerous Cell Membranes

**DOI:** 10.1021/acs.langmuir.4c03948

**Published:** 2024-12-12

**Authors:** Ellen C. Wrobel, Lucas Stori de Lara, Ângelo de Fátima, Osvaldo N. Oliveira

**Affiliations:** † Sao Carlos Institute of Physics, University of Sao Paulo, CP 369, 13560-970 São Carlos, SP, Brazil; ‡ Department of Physics, State University of Ponta Grossa, 84030-900 Ponta Grossa, PR, Brazil; § Department of Chemistry, Institute of Exact Sciences, Federal University of Minas Gerais, 31270-901 Belo Horizonte, MG, Brazil

## Abstract

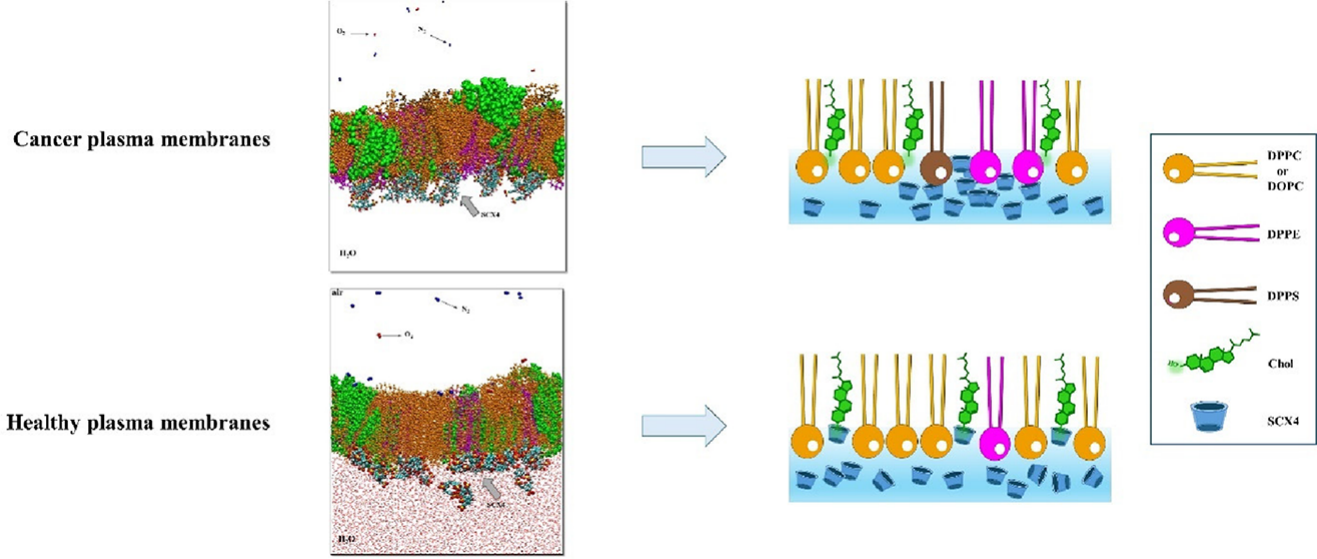

The design of chemotherapeutic drug carriers requires precise information on their interaction with the plasma membrane since the carriers should be internalized by cells without disrupting or compromising the overall integrity of the membrane. In this study, we employ Langmuir monolayers mimicking the outer leaflet of plasma membranes of healthy and cancerous cells to determine the molecular-level interactions with a water-soluble calixarene derivative, *p*-sulfonic acid calix[4]arene (SCX4), which is promising as drug carrier. The cancer membrane models comprised either 40% 1,2-dipalmitoyl-*sn*-glycero-3-phosphocholine (DPPC) or 1,2-dioleoyl-*sn*-glycero-3-phosphocholine (DOPC), 30% cholesterol (Chol), 20% 1,2-dipalmitoyl-*sn*-glycero-3-phosphoethanolamine (DPPE), and 10% 1,2-dipalmitoyl-*sn*-glycero-3-phospho-l-serine (DPPS). The healthy membrane models were composed of 60% DPPC or DOPC, 30% Chol, and 10% DPPE. SCX4 expanded the surface pressure isotherms and decreased compressional moduli in all membrane models, altering their morphologies as seen in Brewster angle microscopy images. A combination of polarization-modulated infrared reflection absorption spectroscopy and molecular dynamics simulations revealed that SCX4 interacts preferentially with lipid headgroups in cancer membrane models through electrostatic interactions with the amine groups of DPPS and DPPE. In healthy membrane models, SCX4 interacts mostly with cholesterol through van der Waals forces. Using a multidimensional projection technique to compare data from the distinct membrane models, we observed that SCX4 effects depend on membrane composition with no preference for cancer or healthy membrane models, which is consistent with its biocompatibility. Furthermore, the interactions and close location of SCX4 to the headgroups indicate that it does not compromise membrane integrity, confirming that SCX4 may be a suitable drug carrier.

## Introduction

Calixarenes are oligomers synthesized through condensation of para-substituted phenols with formaldehyde in the presence of strong acid or alkaline catalysts.^1−3^ These molecules are shaped like truncated cones with phenolic units linked by methyl bridges at the ortho position to the hydroxyl group. As representers of the third generation of supramolecular chemistry,^3,4^ calixarenes may have steric flexibility, hydrophobic cavities of variable sizes, biocompatibility, inertness, and nontoxicity.^5^ The water-soluble *p-*sulfonatocalix[*n*]arenes are notable for their thermal and chemical stability, high solubility in aqueous media, biocompatibility, nontoxicity, and lack of hemolytic activity. These features make them suitable for drug delivery vehicles,^6^ as inclusion complexes,^7^ micelles,^8^ hydrogels,^9^ liposomes,^10^ and vesicles.^5,11,12^ Calixarenes have been used for anticancer drug delivery with chemotherapeutic agents such as doxorubicin,^13,14^ Temozolomide,^7,15^ curcumin,^16,17^ paclitaxel,^18^ and camptothecin.^19^ Using calixarenes has proven efficient for targeted cytotoxicity against cancer cell lines.

Ideal drug delivery vehicles overcome biological barriers to achieve effective antitumor effects. While their interactions with the cell membrane are required,^13^ maintaining plasma membrane integrity is also crucial.^20^ Preserving membrane integrity with drug carriers mitigates cytotoxicity and maintains cellular function, thereby preventing adverse effects and ensuring safer and more effective drug delivery.^21^ Since the cell membrane serves as a barrier for drugs and drug carriers, it is important to determine the molecular-level interactions between nanocarriers, such as calixarene derivatives, and the cell membrane. Despite the inherent complexity of cell membranes, their primary structure is composed of phospholipids. This fundamental characteristic allows for studying the interactions using phospholipid membrane-mimicking systems, such as Langmuir monolayers.^22−30^ Although these monolayers mimic only one leaflet of a cell membrane, they are useful due to the ability to control composition and surface packing.^22,28,31,32^ Additionally, a key application of studying Langmuir monolayers is in understanding how biologically relevant molecules interact with membranes. They help assess whether these molecules penetrate the membrane, how they influence membrane properties, and which functional groups may be involved in the interactions.^28^ Interactions between lipid monolayers and calixarenes have been investigated mostly for distinguishing effects on bacterial and human membrane models. For instance, the impact of calixarenes has been determined on lipid monolayers such as 1,2-dimyristoyl-*sn*-glycero-3-phosphoethanolamine (DMPE),^33−35^ 1,2-dimyristoyl-*sn*-glycero-3-phosphocholine (DMPC),^35,36^ 1,2-dimyristoyl-*sn*-glycero-3-phospho-*rac*-(1-glycerol) (DMPG),^35^ and 1,2-dimyristoyl-*sn*-glycero-3-phospho-l-serine (DMPS),^35,36^ cholesterol,^37^ 1,2-dilauroyl-*sn*-glycero-3-phosphocholine (DLPC),^38^ and 1,2-dipalmitoyl-*sn*-glycero-3-phospho-(1′-*rac*-glycerol) DPPG^39^ lipid monolayers.

**Figure 1:**
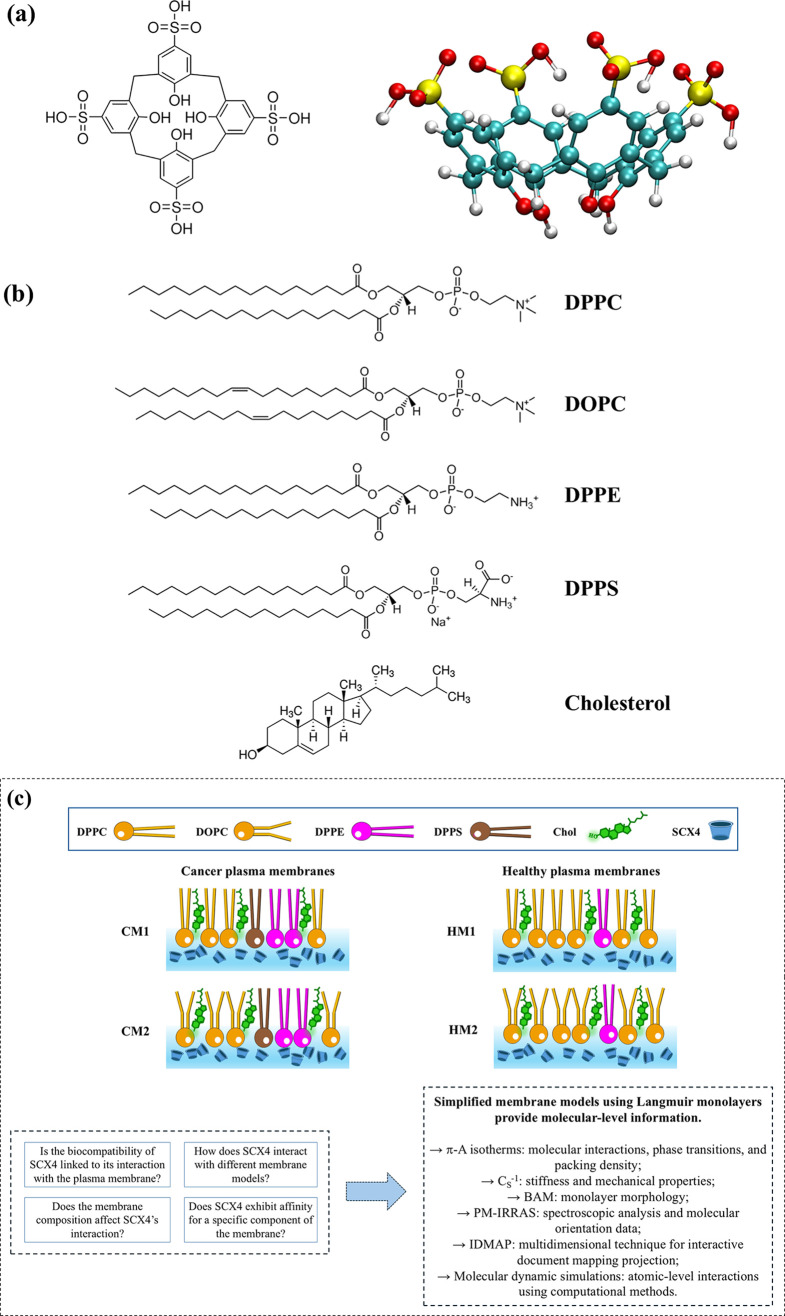
(a) Molecular structures for the *p*-sulfonic acid calix[4]arene. Oxygen atoms–red, carbon atoms–blue, hydrogen atoms–white, and sulfur atoms–yellow; (b) Molecular structures of the membrane model compounds DPPC, DOPC, DPPE, DPPS, and cholesterol; (c) Scheme illustrating the study outline.

In this paper, we investigate the interaction of a water-soluble calixarene derivative, *p*-sulfonic acid calix[4]arene (SCX4), with simplified models of cancerous and healthy cell membranes. The main aim is to determine if SCX4 exhibits a preference for specific membrane components and whether these interactions relate to its known biocompatibility. The latter comprises Langmuir monolayers of saturated and/or unsaturated lipids and cholesterol. Figure 1 provides a visual overview of the objectives in this study. The interactions were explored using surface pressure–area (π–*A*) isotherms, Brewster angle microscopy (BAM), and polarization-modulated infrared reflection absorption spectroscopy (PM-IRRAS). Considering the diverse effects of varying concentrations of SCX4 across distinct membrane models, we employ a multidimensional projection technique^40^ to analyze the π–*A* isotherms and PM-IRRAS data. We complemented experimental techniques with molecular dynamics (MD) simulations, which allowed us to determine the precise location and molecular-level interactions involving SCX4 in the membranes.

## Experimental Details

### Materials

The *p*-sulfonic acid calix[4]arene derivative (SCX4) was synthesized and purified through a well-established route^41^ The phospholipids 1,2-dioleoyl*-sn-*glycero-3-phosphocholine (DOPC), 1,2-dipalmitoyl-*sn*-glycero-3-phosphocholine (DPPC), 1,2-dipalmitoyl-*sn*-glycero-3-phosphoethanolamine (DPPE), and 1,2-dipalmitoyl-*sn*-glycero-3-phospho-l-serine (DPPS) were obtained from Avanti Polar Lipids Inc. Cholesterol (Chol) was purchased from Sigma-Aldrich. The chemical structures of the SCX4, phospholipids, and cholesterol are depicted in Figure 1. Chloroform, employed as the spreading solvent in Langmuir monolayer experiments, was of HPLC grade and purchased from Panreac. Methanol was acquired from Synth. The salts NaCl, KCl, Na_2_HPO_4_, and KH_2_PO_4_ employed for preparing the phosphate-buffered saline solution (PBS, pH 7.4) were purchased from Synth, Neon, or Exodo Científica (Brazil). Ultrapure water (resistivity, 18.2 MΩ cm, pH 5.6 at 22 ± 1 °C) was obtained from a Millipore Milli-Q system.

### Langmuir Monolayers

The experiments were carried out with a custom-built Langmuir trough (surface area, 159 cm^2^) connected to a KSV NIMA instrument (KSV 5000, KSV Instruments, Ltd., Helsinki, Finland). The trough and its barriers were made of Teflon. The trough was equipped with a filter paper Wilhelmy plate (Whatman Chr1) for surface pressure measurements and an electrobalance with a resolution of 4 μN/m. The definition of surface pressure (π) is provided by eq 1:
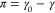
1where γ_0_ represents the surface tension of water in the absence of the monolayer and γ is the surface tension due to the monolayer. Before each experiment, the Langmuir trough was cleaned with ethanol, chloroform, and ultrapure water, followed by filling with 65 mL of phosphate-buffered saline (PBS, pH 7.4) prepared according to the Cold Spring Harbor protocols.^42^ The spreading solutions at 1 × 10^–3^ mol/L were prepared by dissolving DPPC, DOPC, and Chol in chloroform, and DPPE and DPPS in chloroform:methanol mixture (4:1 and 3:2 v/v), respectively.

Our study focuses on mimicking the mammalian plasma cell membrane, which primarily consists of glycerolipids (65% mol), sphingolipids (10% mol), and cholesterol (25% mol).^43−45^ The lipid compositions for healthy (HM1 and HM2) and cancerous (CM1 and CM2) plasma membranes were selected based on established literature^43,44,46,47^ and prior research findings.^27^ Phosphatidylcholine, a zwitterionic lipid, is the predominant phospholipid in the outer leaflet of healthy cell membranes.^43,48,49^ Phosphatidylethanolamine and phosphatidylserine are typically located in the inner leaflet but shift to the outer leaflet in many cancers.^43,48,50^ This translocation makes both lipids useful as cancer biomarkers. Cholesterol plays a critical role for maintaining membrane structural integrity and fluidity^51,52^ While sphingomyelin is a notable component of plasma membranes^45^ our study focused on glycerolipids and cholesterol interactions with the calixarene derivative, with potential for future studies to explore sphingomyelin interactions. Our investigation featured two types of membrane systems: one with only saturated lipids (HM1 and CM1), which provide rigidity and stability, and another with the unsaturated lipid DOPC (HM2 and CM2), which enhances fluidity and flexibility.^53^ Specifically, the cancer membrane models included either DPPC or DOPC, 30% Chol, 20% DPPE, and 10% DPPS. The healthy membrane models consisted of 60% DPPC or DOPC, 30% Chol and 10% DPPE. These compositions are illustrated in Figure 1c. Notably, models CM2 and HM2 incorporate DOPC, which is depicted in the figure with kinks in the tails due to its unsaturated bonds, distinguishing them from CM1 and HM1 models, which contain only saturated lipids. This selection reflects the asymmetric lipid composition of the outer leaflet, providing a comprehensive basis for understanding membrane behavior in healthy and cancerous cells.

CM1, HM1, CM2, and HM2 mixed monolayers were obtained by mixing appropriate volumes of stock solutions of lipids and cholesterol prior to cospreading them at the air–water interface. The aqueous subphase consisted of PBS buffer (pH 7.4) in the absence and in the presence of *p-*sulfonic acid calix[4]arene (SCX4). A stock SCX4 solution (1 × 10^–4^ mol/L) was prepared by dissolving SCX4 in a PBS buffer solution and stirring in an ultrasonic bath for 1 h. This stock solution was utilized to obtain the other concentrations (1 μM, 10 μM, and 30 μM). Pure lipids and cholesterol, HM1, HM2, CM1, and CM2 membrane models were spread onto the PBS subphase, with or without SCX4, using a Hamilton microsyringe. Following 15 min of solvent evaporation, the barriers were symmetrically compressed at a rate of 10 mm/min. The Wilhelmy method was employed to measure the surface pressure. All experiments were carried out at 22 ± 1 °C. Surface pressure versus mean molecular area (π–*A*) measurements were conducted in triplicate.

To evaluate the mechanical properties of the monolayers in the presence and absence of SCX4, we calculated the surface compressional modulus (*C*_S_^–1^) from the π–*A* isotherms, as per eq 2:
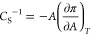
2where *A* represents the average molecular area at a given surface pressure π. *C*_S_^–1^ values below 25 mN/m imply a low-density liquid phase, while those within the range of 25–50 mN/m denote a liquid-expanded (LE) state. A range of 100–250 mN/m suggests a liquid-condensed (LC) state, and values above 500 mN/m indicate a solid-state film.^28^ For noise reduction, the raw compressibility curves were smoothed using an adjacent-averaging method, with a 10-point window.

The π–*A* isotherms and *C*_S_^–1^ data of each membrane model, pure lipids, and cholesterol on a PBS subphase with or without SCX4 were examined using the multidimensional interactive document mapping (IDMAP) projection technique,^40^ via software PEx-Sensors.^54^ This dimensionality reduction technique has demonstrated strong effectiveness in sensing applications,^54^ but recently our group has extended the use of IDMAP to examine interactions within Langmuir monolayers.^27,29,30,55^ This technique enabled straightforward comparison of the different mixed systems by converting the whole π–*A* isotherms or *C*_S_^–1^ curve^55^ into single-colored dots on a map through the *Fastmap* dimension reduction algorithm.^56^ The IDMAP projection works by calculating Euclidean distances (δ) between data points in the original high-dimensional space *X* = {*x*_1_, *x*_2_, ..., *x*_*n*_} and mapping them onto a lower-dimensional space *Y* = {*y*_1_, *y*_2_, ..., *y*_*n*_}, where the function *f*_IDMAP_ minimizes differences between distances in both spaces. This is represented by eq 3:
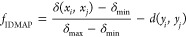
3where δ(*x*_*i*_, *x*_*j*_) represents the Euclidean distance between two data instances *x*_*i*_ and *x*_*j*_ in the original space, δ_min_ and δ_max_ are the minimum and maximum Euclidean distances between any two data points in this original space, which are used to normalize distances for consistency in the mapping process, and *d*(*y*_*i*_, *y*_*j*_) represents the Euclidean distance between the projected points *y*_*i*_ and *y*_*j*_ on the 2D space.^54^ π–*A* isotherms and *C*_S_^–1^ data within the range of 0 to 35 mN/m are depicted as single data points projected onto a 2D Euclidean space, where close proximity indicates similarity based on Euclidean distances. Silhouette coefficient (*S*) values, ranging from −1 to 1, were utilized to evaluate the quality of projection, with higher values indicating superior data discrimination.^54^ Relative Euclidean distances from the maps were determined using ImageJ software to explore the impact of varying SCX4 concentrations on the membrane models.

Brewster angle microscopy (BAM) serve as the method for real-time visualization of the different membrane models and the impact of SCX4 on them. Images were captured using an ellipsometer Accurion EP4 (Accurion, Göttingen, Germany) coupled to a KSV Nima Langmuir trough. This setup featured a 50 mW laser and a polarizer emitting light at a wavelength of 658 nm with 100% power and *p*-polarized characteristics. Reflected light was captured by a high-quality ultraobjective camera, SVS-Vistek eco 285. Throughout the measurements, the incident angle remained constant at 53.1°, with the polarizer, analyzer, and compensator set to 2.0, 10.0, and 0.0°, respectively. Image correction was performed using DataStudio software, adjusting greyscale to address excessive brightness. Additionally, contrast and brightness were modified in ImageJ software to enhance the visualization of structures within the images. Monolayers were compressed at a rate of 10 mm/min, with images acquired at several surface pressures along the monolayers formation.

Polarization-modulated infrared reflection–absorption spectroscopy (PM-IRRAS) experiments were conducted using a KSV PMI 550 Instrument (KSV Instruments, Ltd., Helsinki, Finland) installed on a Mini KSV Langmuir setup, with polarization alternating between *p* and *s* at a high frequency. The incidence angle was fixed at 81°. Each spectrum comprised 600 scans with a spectral resolution of 8 cm^–1^. The spectra were taken for monolayers of the pure lipids, cholesterol, healthy and cancerous membrane models on a PBS subphase, in the absence and presence of 30 μM of SCX4 at 30 mN/m. To ensure reproducibility, each experiment was repeated at least twice.

### Classical Molecular Dynamics

MD calculations were conducted using the Large Atomic–Molecular Massively Parallel Simulator (LAMMPS) package.^57^ Initially, atomistic interactions within each component of the systems were individually modeled. This involved separate calculations for the water solution, air (N_2_–O_2_ gas), membrane models, and SCX4 molecules. The membrane models were configured following the experimental conditions, with the healthy membrane model (HM) comprising 60% DPPC or DOPC, 30% cholesterol, and 10% DPPE, while the cancerous membrane model (CM) consisted of 40% DPPC or DOPC, 30% cholesterol, 20% DPPE, and 10% DPPS. Intermolecular and intramolecular potentials were considered for different molecules, with flexibility influenced by the arrangement of charge distributions and binding energies. Analogous to the differentiation between saturated and unsaturated lipids—due to the presence of double versus single bonds—computer simulations incorporated distinct binding energy values for these bond types. Subsequently, the systems were further modeled to include interfaces between materials, incorporating biphasic interactions such as air/(water + SCX4), (water + SCX4)/HM, and (water + SCX4)/CM, as well as triphasic interactions like air/HM/(water + SCX4) and air/CM/(water + SCX4). This allows us to monitor surface pressure in the different models and compare them with the experimental results.

The SPCE–FH model^58^ was employed to describe force fields between water molecules in the water solution, as well as water–air (N_2_–O_2_) interactions. A sample comprising 80,000 water molecules, 152 nitrogen (N_2_) molecules, and 46 oxygen (O_2_) molecules was utilized to simulate the water and air phases, respectively. The air phase was modeled using two Lennard–Jones sites connected by a rigid bond, as detailed by Jiang et al.^59^ The molecular geometry of the SCX4 molecule was initially preoptimized using the CVFF force field without partial charges. Subsequently, partial charges were assigned using the AM1 semiempirical method. This preoptimized molecular geometry was further optimized using CVFF.^60,61^ For the molecules constituting the membrane models (phospholipids and cholesterol), MD calculations were performed using the CHARMM27 force field.^62^ This process was repeated until the convergence criterion, truncated at 0.001 kcal Å^–1^ mol^–1^, was achieved. In addition to the individual components (air, water, SCX4, lipids, and cholesterol), planar interfaces were simulated as described previously. These systems were simulated with a box set to dimensions *L*_*x*_ × *L*_*y*_ × *L*_*z*_, where *L*_*x*_ = 18.0 nm, *L*_*y*_ = 18.0 nm, and *L*_*z*_ = 23.0 nm.

A sequence of simulations in the *NVE* (microcanonical ensemble), *NVT* (canonical ensemble), and *NPT* (isothermal–isobaric ensemble) were performed to attain equilibrium thermodynamic properties for each system at 300 K and 1 atm. At this calculation level, for each system a 1.0 ps run was performed in the *NVE* ensemble, a 10.0 ps run in the *NVT* ensemble, and a 60.0 ns run in the *NPT* ensemble. Periodic boundary conditions were applied throughout. For long-range electrostatic interactions, the reciprocal-space particle–particle particle-mesh (PPPM) method^63−65^ was adopted.

**Figure 2:**
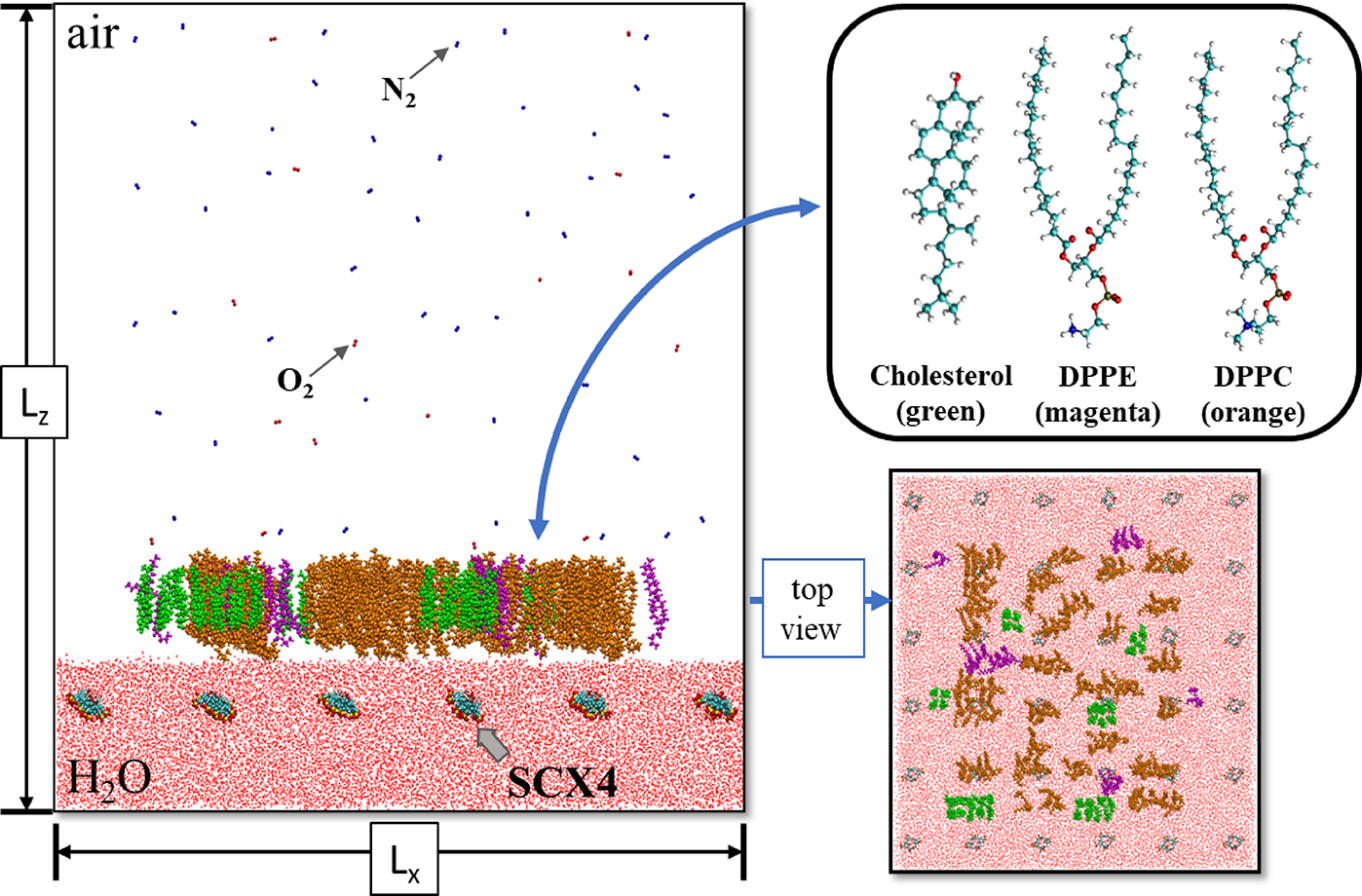
Initial position (after optimization for the isolated systems) and box dimensions (*L*_*x*_ = 18.0 nm and *L*_*z*_ = 23.0 nm) for triphasic (water + SCX4)–(HM1)–air interface; where HM1 comprises 60% DPPC, 30% cholesterol, and 10% DPPE.

Following the investigation of biphasic systems, we proceeded to simulate the triphasic system interfaces, specifically the air/HM/(water + SCX4) and air/CM/(water + SCX4). Figure 2 shows the molecular geometry of the air/HM1/(water + SCX4)–(HM1) interface. A similar procedure was followed for the studies of HM2, CM1, and CM2 monolayers. Another procedure for equilibrating the thermodynamic parameters involved sequential stages of 1.0 ps in the *NVE* ensemble, 50.0 ps in the *NVT* ensemble, and 20.0 ps in the *NPT* ensemble simulations. Then, MD runs were performed for 100.0 ns within the *NVT* ensemble to acquire the results. Throughout these calculations, a time step of 0.5 fs and a cutoff of 12.0 Å were employed for van der Waals interactions. The temperature (300 K) and pressure were controlled with a Nose–Hoover thermostat and an Andersen barostat, respectively. The Lorentz–Berthelot mixing rules were employed to combine the intermolecular potential parameters between mixed systems.^66^

After achieving equilibrium across all systems, we carried out the compression processes at the interfaces to simulate experimental surface pressure isotherms. Surface tensions at the water/air interface (γ_water/air_) were calculated using the Gibbs formulation^67^ for interfacial tension (eq 4), applicable to both bi- and triphasic models. This formulation involves pressure terms where *L*_*a*_ and −*L*_*b*_ define the boundaries of the interfacial region, with *p*_*ab*_(*z*) and *p*_*T*_(*z*) representing the tangential and normal components of the pressure tensor, respectively. Surface pressure values of π = 0, 1, 5, and 10 mN/m were considered. We examined surface pressures exceeding 10 mN/m, yet no further changes were observed in the interactions upon increasing the pressure on the systems. The organization and interactions observed at 10 mN/m were similar to those obtained at 30 mN/m, which is in the biologically relevant pressure regime^68^
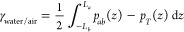
4

**Figure 3:**
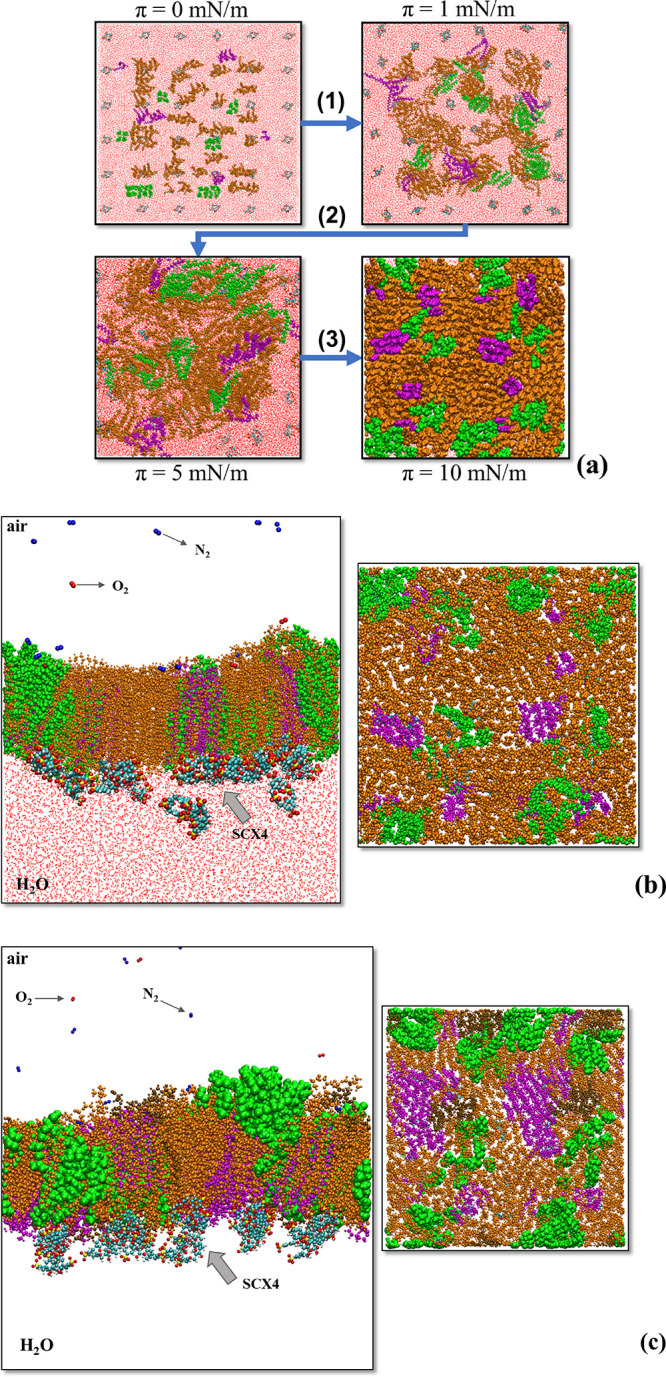
Computational workflow for analyzing the structural properties of the investigated interfaces: (a) Top view of the HM1 monolayer on a water subphase containing SCX4, detailing the following steps: (1) Every 10.0 ns: 0.5 ns for volume deformation; 8.0 ns for thermodynamic stabilization, and 1.5 ns for obtaining the structural properties; (2) Replication of procedures at different surface pressures; (3) Acquisition of the interface at 10 mN/m. Lateral and top views of the (b) HM1 and (c) CM1 monolayers on a water subphase containing SCX4 at 10 mN/m; and (c) Lateral and top view of the CM1 monolayer on water containing SCX4 at 10 mN/m. DPPC, cholesterol, DPPE, and DPPS molecules are represented in orange, green, magenta, and brown, respectively.

The volume dimensions were adjusted to create a new interfacial area. During the results acquisition phase, which lasted 100.0 ns with 10.0 ns intervals, we performed compression and stabilization processes within the systems. This involves 0.5 ns for volume deformation, 8.0 ns for stabilizing thermodynamic parameters, and 1.5 ns for obtaining structural parameters like volumetric and linear density profiles, as well as the radial distribution function *g*(*r*). Figure 3 outlines this simulation procedure. To ensure the accuracy and reliability of our results, each simulation involving surface pressures and volumetric density analyses was conducted with a detailed and systematic approach. For each surface pressure condition, the system was first equilibrated to achieve a stable state. Only after this equilibration were the volumetric density analyses performed. This methodology ensured that the data used for analysis were collected after the system had fully equilibrated, rather than from isolated time points. The figures presented show molecule and domain positions at specific instances, but these snapshots represent final configurations achieved after complete equilibration of the thermodynamic parameters. As a result, the data are statistically reliable and accurately reflect the equilibrated state.

## Results and Discussion

**Figure 4:**
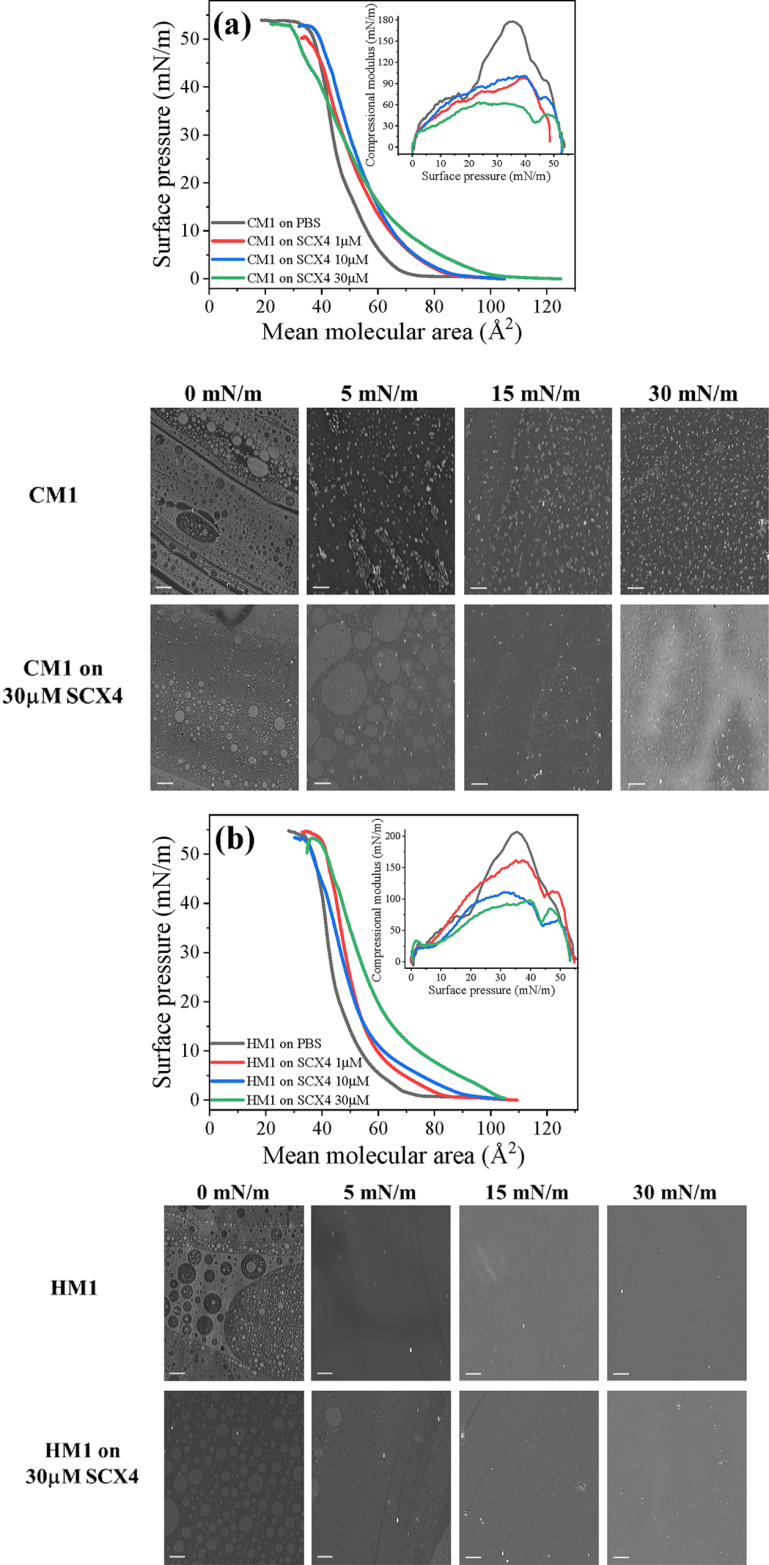

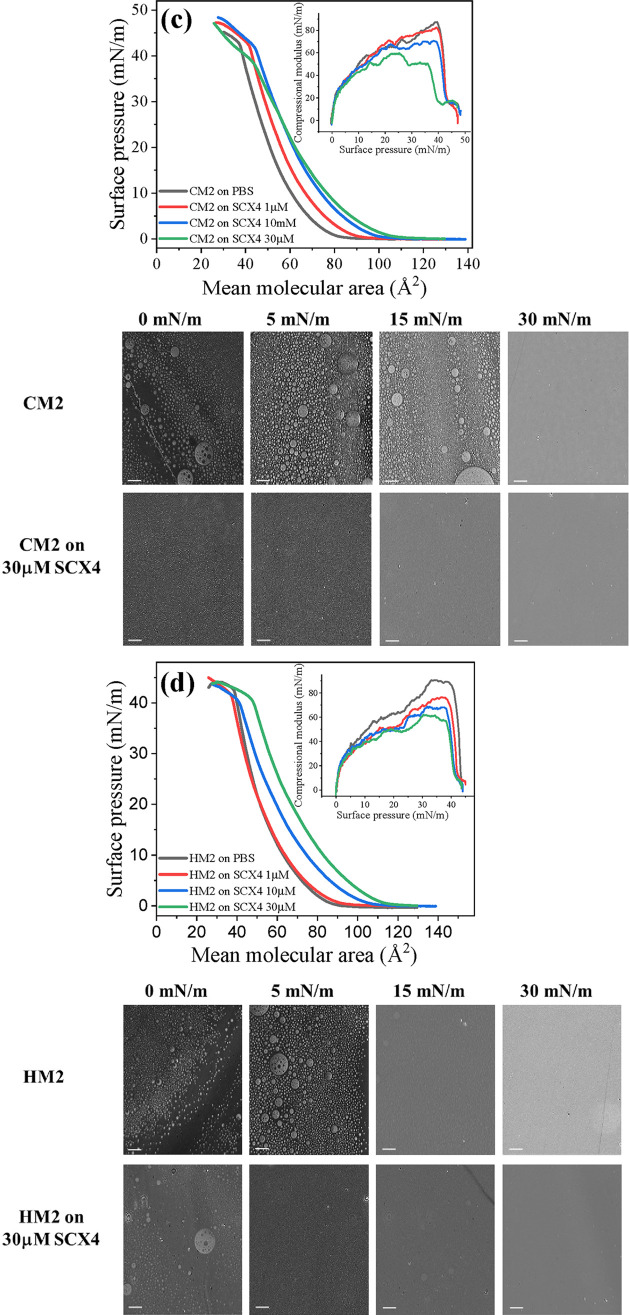
Surface pressure–area (π–*A*) isotherms, compressional moduli (*C*_S_^–1^), and BAM images (the scale bar represents 50 μm) for monolayers of (a, c) cancerous (CM1 and CM2) and (b, d) healthy (HM1 and HM2) membrane models on PBS subphase containing 0, 1, 10, and 30 μM of SCX4.

The plasma membrane is the primary barrier for intracellular delivery of macromolecules, governing trafficking between the interior and exterior of cells.^69^ Herein, we tailored the lipid composition of DPPC, DOPC, DPPE, and DPPS, and cholesterol to mimic both healthy (HM1 and HM2) and cancerous (CM1 and CM2) plasma membranes, based on previous research findings.^27^ The surface pressure–area (π–*A*) isotherms and compressional moduli data (*C*_S_^–1^) for pure lipids and cholesterol in Figure S1 are consistent with those in the literature.^70−77^ The limiting area (A_0_) was determined by extrapolating a tangent line from the pressure–area isotherm of the more ordered phase to zero pressure. For DPPC, DOPC, DPPE, DPPS, and Chol, the respective values for A_0_ and the maximum *C*_S_^–1^ are 57 Å^2^ and 210 mN/m, 83 Å^2^ and 75 mN/m, 51 Å^2^ and 250 mN/m, 54 Å^2^ and 185 mN/m, and 41 Å^2^ and 380 mN/m. The π–*A* isotherms and compressional moduli for monolayers representing cancerous cell membranes (CM1 and CM2) and healthy cell membranes (HM1 and HM2) are illustrated in Figure 4, whose key features are summarized in Table 1. The limiting areas (A_0_) for CM1, HM1, CM2, and HM2 are 53.4, 50.1, 63.3, and 62.4 Å^2^, respectively. The π–*A* isotherms lay between those of pure DPPC (or DOPC for CM2 and HM2) and cholesterol, the major components in these models. Additionally, the inclusion of cholesterol results in the disappearance of the liquid-expanded/liquid-condensed (LE-LC) phase transition typically observed in pure DPPC.^78,79^ The maximum surface compressional moduli closely resemble those of pure DPPC (in CM1 and HM1 models) and DOPC (in CM2 and HM2 models) lipids, given their predominance in the mixed systems. The maximum *C*_S_^–1^ for CM1, HM1, CM2, and HM2 is 177, 208, 87, 91 mN/m, respectively. The smaller *C*_S_^–1^ in CM2 and HM2 are attributed to the incorporation of a double unsaturated lipid, DOPC, which increased monolayer fluidity and flexibility. Although the literature suggests that cholesterol typically condenses lipid monolayers, especially at higher contents,^78−80^ we did not observe a significant condensation when using a 30% cholesterol content. Our findings align with those of Telesford et al.,^81^ who found that low molar ratios of cholesterol (0.1–0.3) do not affect the compressional modulus of DPPC significantly. At a biologically relevant pressure (π ∼ 30 mN/m),^68^ the respective mean molecular area and *C*_S_^–1^ are 44.0 Å^2^ and 152 mN/m, 42.8 Å^2^ and 173 mN/m, 44.0 Å^2^ and 71 mN/m, 44.1 Å^2^ and 79 mN/m for CM1, HM1, CM2, and HM2 models.

**Table 1:** Parameters for the Monolayers of Cancer (CM1 and CM2) and Healthy (HM1 and HM2) Membrane Models on PBS Subphase Containing 1, 10, and 30 μM SCX4^a^

	*A*_ex_ (Å^2^)	*A*_30_ (Å^2^)	π_col_ (mN/m)	*C*_S_^–1^_max_ (mN/m)	*C*_S_^–1^_30_ (mN/m)
CM1 on PBS	53.4 ± 0.1	44.0 ± 0.1	52.7 ± 0.3	177 ± 5	152 ± 5
CM1 on SCX4 1 μΜ	63.6 ± 0.5	47.4 ± 0.3	50.0 ± 0.4	100 ± 2	82 ± 2
CM1 on SCX4 10 μΜ	66.6 ± 0.4	49.9 ± 0.2	52.1 ± 0.3	102 ± 1	93 ± 3
CM1 on SCX4 30 μΜ	73.3 ± 1.3	47.3 ± 0.4	52.6 ± 0.2	63 ± 3	60 ± 2
HM1 on PBS	50.1 ± 0.6	42.8 ± 0.3	53.8 ± 0.4	208 ± 3	173 ± 2
HM1 on SCX4 1 μΜ	58.9 ± 0.2	47.8 ± 0.3	53.5 ± 0.4	161 ± 4	145 ± 2
HM1 on SCX4 10 μΜ	61.0 ± 0.4	46.6 ± 0.1	52.9 ± 0.3	110 ± 7	108 ± 3
HM1 on SCX4 30 μΜ	72.2 ± 1.5	52.6 ± 0.4	52.6 ± 0.4	98 ± 5	88 ± 3
CM2 on PBS	63.3 ± 0.4	44.0 ± 0.3	42.6 ± 0.3	87 ± 7	71 ± 2
CM2 on SCX4 1 μΜ	71.2 ± 0.2	48.8 ± 0.1	42.3 ± 0.5	82 ± 2	75 ± 3
CM2 on SCX4 10 μΜ	77.6 ± 0.4	52.6 ± 0.1	42.6 ± 0.2	67 ± 2	66 ± 2
CM2 on SCX4 30 μΜ	82.4 ± 0.4	51.9 ± 0.6	39.9 ± 0.5	60 ± 1	50 ± 2
HM2 on PBS	62.4 ± 0.6	44.1 ± 0.4	42.8 ± 0.2	91 ± 1	79 ± 3
HM2 on SCX4 1 μΜ	63.7 ± 0.3	43.4 ± 0.3	41.7 ± 0.2	76 ± 3	69 ± 1
HM2 on SCX4 10 μΜ	74.8 ± 0.8	48.3 ± 0.4	40.3 ± 0.3	68 ± 2	63 ± 2
HM2 on SCX4 30 μΜ	82.4 ± 0.3	55.8 ± 0.3	41.5 ± 0.5	61 ± 2	62 ± 3

a Abbreviations: *A*_ex_, extrapolated area; *A*_30_, molecular area at 30 mN/m; π_col_, collapse pressure; *C*_S_^–1^_max_, maximum compressional modulus; *C*_*S*_^–1^_30_, compressional modulus at 30 mN/m.

To investigate whether biocompatibility and effectiveness as a carrier of SCX4 are related to its action on cell membranes, we employed Langmuir monolayers to determine these molecular-level interactions. The effect of different concentrations of SCX4 (1, 10, and 30 μM) on the cancer and healthy plasma membrane models is illustrated in Figure 4, with the main parameters in Table 1. The effect from SCX4 on the pure phospholipids and cholesterol used in the membrane models was compared in a systematic study whose results are shown in the Supporting Information (see Figures S2 and S3 and Table S1). The incorporation of SCX4 expanded the π–*A* isotherms across all examined membrane models, except for the HM2 monolayer on PBS subphase containing 1 μM SCX4, which were accompanied by a decrease in compressional moduli. The impact of SCX4 was more pronounced in membrane models containing solely saturated lipids (CM1 and HM1). This resulted not only in a shift in the molecular area but also in alterations to the shape of the isotherms, particularly at high SCX4 concentrations. Conversely, in models with both saturated and unsaturated lipids (CM2 and HM2), SCX4 incorporation primarily resulted in an expansion of molecular areas without significant change in isotherm shape. Moreover, the influence of SCX4 on compressional modulus was more pronounced in CM1 and HM1 models, exhibiting a decrease in *C*_S_^–1^ at 30 mN/m of approximately 60% and 47%, respectively, with 30 μM SCX4 in the subphase. For CM2 and HM2 models, this decrease was approximately 28% and 19%, respectively. These differences in the effect of SCX4 on the mechanical properties of model membranes can be related to their composition. CM2 and HM2 exhibit greater fluidity and less compact packing due to DOPC unsaturated lipids. In contrast, SCX4 incorporation in CM1 and HM1 monolayers affects their mechanical properties more significantly, as their stiffness is easily disrupted. This suggests that while CM2 and HM2 monolayers do interact with SCX4 molecules, their compressional modulus could be less affected.

The effects of SCX4 on the membrane models were confirmed by plotting the π–*A* isotherms and *C*_S_^–1^ data using the IDMAP multidimensional projection technique (Figure S4). Analyses were conducted for isotherms ranging from 0 to 35 mN/m, with each isotherm and *C*_S_^–1^ curve represented by a colored dot on a 2D map. The IDMAP technique allows for a comparison of entire isotherms or specific regions, enabling us to observe the distinct effects of SCX4 on the monolayers beyond merely comparing specific molecular areas, collapse pressures and compressional moduli.^55^ The IDMAP analysis reveals that SCX4 induced substantial changes in all π–*A* isotherms of the membrane models (Figure 4a), even at the lowest SCX4 concentration, with the associated data points being far apart. The silhouette coefficient (*S*) was 0.72, indicating a meaningful level of data discrimination.^82^ The relative Euclidean distances in the inset of Figure S4a between isotherms with and without SCX4 in the subphase allow for direct comparison of the induced effects. A greater separation of the clusters is observed as the SCX4 concentration increases, with a more pronounced effect for HM1 and HM2 monolayers on a PBS subphase containing 30 μM SCX4. Notably, the presence of SCX4 induces a separation in the vertical plane of the map, with membrane models on the PBS subphase positioned on the left side and those on PBS containing SCX4 subphases on the right side. Regarding the effect of SCX4 on the *C*_S_^–1^ data in Figure S4b, there is a reasonable level of data discrimination with a silhouette coefficient of 0.52, though not as high as for the π–*A* isotherms. This lower discrimination is attributed to the weaker effect of SCX4 on membrane models containing an unsaturated lipid (HM2 and CM2). Due to the higher fluidity of these models, their *C*_S_^–1^ values are less affected by the incorporation of SCX4 compared to models containing only saturated lipids. The highest separation of clusters was observed for the CM1 membrane model on a subphase containing 30 μM SCX4. Although the HM2 and CM2 did not exhibit a significant separation of clusters, there remained some distinction (see Figure S5) when analyzing only these systems using the IDMAP technique, with a silhouette coefficient of 0.42.

The analysis of Brewster angle microscopy images in Figure 4 confirmed that incorporating SCX4 into both the cancer and healthy models led to a noticeable change in their morphology. In the CM1 monolayer at 0 mN/m, 2D foam-like structures^83,84^ are observed, suggesting an equilibrium between gaseous and liquid phases, typical of high mean molecular areas at the early stages of monolayer formation. At 5 mN/m, bright LC domains with ∼10 μm in diameter coexist with a LE phase (darker homogeneous background), typical of mixed lipid-cholesterol systems.^85−88^ With further compression, the number of these domains increases until reaching the collapse point. Notably, these bright domains are not observed in similar monolayers lacking DPPS (as seen in the HM1 model discussed below). For the CM1 model, the monolayer is not homogeneous across all compression stages. The appearance of bright spots may suggest limited cholesterol solubility within the phospholipid matrix, which in general is lower in phosphatidylserines than in phosphatidylcholines,^85,89^ leading to monolayer destabilization and then 3D cholesterol crystallites are expelled from the monolayer.^85,87^ The presence of SCX4 altered this morphology; the typical 10 μm LC domains appeared at higher pressures and in reduced numbers. At surface pressures above 15 mN/m, the monolayers exhibited enhanced homogeneity, with smaller circular LC domains. At 30 mN/m, the monolayer appeared more homogeneous and brighter compared to the system without SCX4. We may suggest that SCX4, due to its high affinity for the polar headgroup of DPPS (as supported by the Molecular Dynamics studies below), interacts strongly with DPPS, thereby preventing the destabilization of monolayers. This DPPS-SCX4 interaction likely reduces the cholesterol-DPPS interactions, leading cholesterol to interact more readily with other phospholipids in the monolayer. Consequently, the expulsion of 3D cholesterol crystallites is reduced, leading to a more homogeneous monolayer structure.

At low surface pressures, the HM1 monolayer initially displays small LC circular domains dispersed within a fluid phase, reflecting the coexistence of liquid-expanded and liquid-condensed phases typical of mixed cholesterol/lipid systems. With further compression, these domains coalesce, resulting in a homogeneous monolayer in the condensed phase. Above 5 mN/m, SCX4 significantly alters the morphology, with numerous bright domains appearing within the condensed monolayer, likely corresponding to cholesterol-rich domains.^27^ Dynamic molecular simulations (described below) suggest that SCX4 in the HM1 model exhibits an affinity for cholesterol domains, which may account for the increased presence of cholesterol crystallites when SCX4 is present. The CM2 and HM2 models exhibit similar morphological characteristics, with circular domains dispersed within a fluid phase at low surface pressures. These domains are more numerous than those observed in the HM1 and CM1 models. As pressure increases beyond 15 mN/m, the domains coalesce, resulting in homogeneous monolayers. The incorporation of SCX4 in the CM2 and HM2 models primarily affects their morphology at low pressures, resulting in smaller circular domains that coalesce at lower pressures compared to systems without SCX4. The interaction between SCX4 and the lipids likely promotes the coalescence of the circular domains. This leads to a more uniform monolayer, reducing the phase separation and enhancing the overall homogeneity of the monolayer. To summarize, SCX4 has a higher impact on CM1 than HM1, but affects similarly HM2 and CM2. This trend is likely due to the presence of the unsaturated lipid DOPC in CM2 and HM2, which tends to form homogeneous monolayers in the LE state, as confirmed by the compressional modulus data.

**Figure 5:**
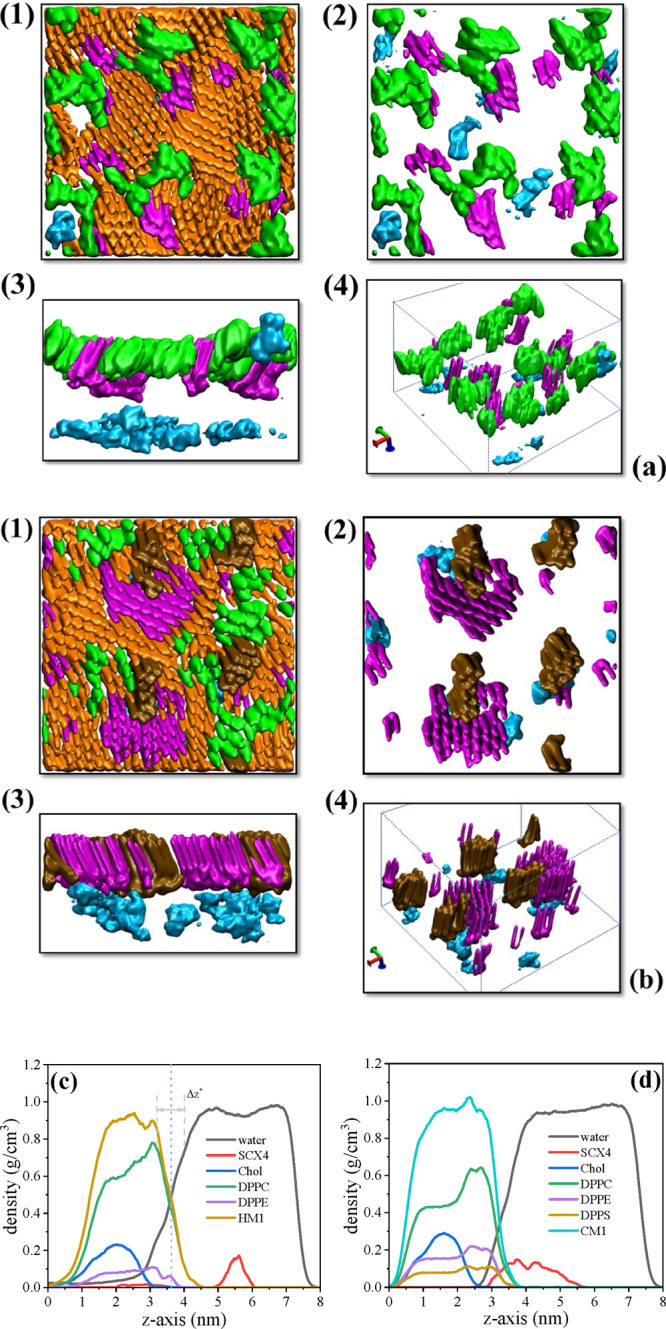
(a) Volumetric density profiles for HM1 monolayers at π = 10 mN/m in different visualizations: (1) top view; (2) top view with DPPC removed; (3) lateral view of (2); (4) 3D view of (2). In these representations, green, orange, magenta, and cyan correspond to cholesterol, DPPC, DPPE, and SCX4 in the monolayer, respectively. (b) Volumetric density profiles for CM1 monolayers at π = 10 mN/m in different visualizations: (1) top view; (2) top view with DPPC removed; (3) lateral view of (2); (4) 3D view of (2). Green, orange, magenta, brown, and cyan represent cholesterol, DPPC, DPPE, DPPS, and SCX4 in the monolayer, respectively. (c, d) Density profiles for (c) (water + SCX4)–(HM1) and (d) (water + SCX4)–(CM1) interfaces. All monolayers were simulated at 300 K and surface pressure π = 10 mN/m. The interface was determined by the Gibbs dividing surface (GDS) and interface region (Δz*) is determined considering the region within the GDS limits.

To elucidate the interactions between SCX4 and the proposed membrane models, we conducted molecular dynamics simulations. Volumetric density profiles were examined using VMD software to evaluate membrane integrity at 10 mN/m. This surface pressure was selected for theoretical investigations since no additional changes were observed in the interactions between the membrane models and SCX4 upon increasing the pressure beyond this value. The volumetric density profile for the HM1 monolayers in Figure 5a reveals that some SCX4 molecules are located within the subphase, while others are situated near the polar region of the membrane. In the latter case, SCX4 molecules align along the membrane, establishing close proximity with cholesterol molecules (refer to the structures in cyan and green in Figure 5a). The preferential interaction with cholesterol is supported by Korchowiec et al.^37^ who studied interactions between two antibacterial calixarene derivatives and cholesterol at the air–water interface. They found that cholesterol promotes dehydration of the calixarene polar groups and facilitates the transfer of the derivatives from the aqueous phase to the gas phase, possibly through hydrophobic interactions with cholesterol. This suggests that cholesterol localized in cell membranes could facilitate transfer of calixarene derivatives across membranes, which would apply to SCX4. In the CM1 cancer membrane model, SCX4 molecules exhibit a preferential interaction with DPPS and DPPE molecules. The volumetric profiles in

Figure 5b reveal a noteworthy finding: SCX4 molecules exhibit closer proximity to CM1 membrane than to the healthy (HM1) membrane. While SCX4 molecules near the surface of the HM1 monolayer remain in the aqueous phase, in CM1 they are accommodated under the domains of DPPS and DPPE molecules. Hence, the membrane composition plays a role in facilitating interactions with SCX4. The presence of DPPS enhances SCX4 proximity to the monolayer, facilitating interactions with other lipids, such as DPPE, with which SCX4 only interacts in the cancer membrane model. This finding suggests that SCX4 may exhibit improved drug delivery properties in cancer cells.

The interactions between SCX4 molecules and HM2 and CM2 models, which contain DOPC unsaturated lipids instead of DPPC, were also investigated via molecular dynamic simulations. Figures S6 and S7 illustrate the volumetric density profiles for these systems, revealing no significant changes compared to the results for CM1 and HM1 systems. This suggests that the fluidity facilitated by unsaturated lipids does not substantially impact the interactions between SCX4 and membrane models. Although theoretical analyses of fluidity and flexibility differences between saturated and unsaturated lipid membranes are valuable, our study focused primarily on understanding how the calixarene derivative interacts with these plasma membrane models. Our findings indicate that SCX4 interactions predominantly occur in the hydrophilic regions of the membrane, implying that membrane fluidity has a minimal impact on these interactions. Instead, it emphasizes that the potential membrane-crossing process for SCX4 molecules is influenced by other factors, namely the polar head of lipids, as evidenced by the altered behavior of SCX4 in the presence of DPPS, along with the presence of cholesterol.

In addition to the volumetric density profile, we analyzed the density profile perpendicular to the water-membrane interface (*z*-axis) of HM1 and CM1 monolayers on an aqueous subphase containing SCX4 molecules. The Gibbs dividing line^90^ (indicated by the dashed line at 3.6 nm on the *z*-axis) separates the air and aqueous phases. For the HM1 system in Figure 5c, DPPC and DPPE molecules and the HM1 monolayer exhibit stronger interactions with water than does cholesterol. As expected, the membrane components are predominantly located in the air phase, with only their polar groups in contact with the aqueous subphase. The SCX4 molecules are situated mainly within the aqueous subphase with its density profile located from 5.0 to 6.0 nm, corresponding to the aqueous phase. SCX4 molecules are also observed within the range of 2.0–3.6 nm (as depicted in the zoomed-in section of Figure 5c, illustrated in Figure S8). This range aligns with the region occupied by DPPC, DPPE, and cholesterol molecules. However, SCX4 molecules exhibit a preference for interacting with cholesterol domains, likely facilitated by hydrophobic interactions. In the density profile along the *z*-axis for the CM1 monolayer (Figure 5d), SCX4 molecules are situated within the range of 3.0 to 5.6 nm, exhibiting a distribution where they are partly localized in the water subphase and partially interacting with the polar groups of the lipids. In this model, the proximity to the lipids is mostly to their polar groups.

We further investigated the interactions between SCX4 molecules and healthy and cancer membrane models using radial pair distribution functions, *g*(*r*). The *g*(*r*) function provides average information about the spatial arrangement of particles and is intrinsically related to interaction potential energies, as outlined in eq 5. In our study, *g*(*r*) profiles were employed to evaluate the distances between SCX4 molecules and key atoms in both healthy and cancer membrane models, allowing us to gain insights into the interactions in both healthy and cancerous membranes.
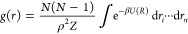
5where *N* is the number of particles, ρ is the particle density, β = 1/*k*_B_*T*, and *U*(*R*) is the interaction potential energy. This relationship demonstrates that *g*(*r*) provides insights into the energy landscape of the system.

In Figure S9a, the *g*(*r*) profiles for the distances from the S atom of SCX4 to the N and P atoms of DPPC and DPPE and to the O atoms of cholesterol, indicate that these atoms are distant from each other. For instance, the first peak for the distance S_SCX4_–N_DPPC_ occurs at 5.4 Å, albeit with a low distribution of *g*(*r*). Similarly, for the distance between S_SCX4_ and N_DPPE_, the first peak is at 3.6 Å. These observations are consistent with the density profile in Figure 5a, where there is a considerable spacing between SCX4 and the HM1 monolayer, with only a minimal number of SCX4 molecules near the monolayer. In contrast to the HM1 monolayer, Figure S9b reveals strong interactions between SCX4 and DPPE and DPPS phospholipids in the CM1 model. A pronounced radial distribution with high intensity was observed for the S atoms of SCX4 and the polar groups of DPPS: S_SCX4_–Na_DPPS_ at 2.4 Å, S_SCX4_–N_DPPS_ at 3.6 Å, and S_SCX4_–P_DPPS_ at 4.2 Å, all exhibiting a radial distribution with high intensity. These distances correspond to SCX4-DPPS interactions. Similarly, for SCX4-DPPE distances, S_SCX4_–N_DPPE_ occurs at 3.8 Å and S_SCX4_–P_DPPE_ at 5.6 Å. In reference to the DPPC and cholesterol components within the CM1 model, the *g*(*r*) profiles in Figure S9c suggest that SCX4 exhibits minimal affinity toward the phosphorus and nitrogen atoms of DPPC, as well as toward the oxygen atoms of cholesterol, as indicated by the low intensities of *g*(*r*). This observation corroborates the volumetric density profile in Figure 5b, providing evidence of the affinities between SCX4 molecules and DPPS and DPPE phospholipids.

The proximity of SCX4 to lipid components in CM models is predominantly driven by electrostatic interactions. Specifically, the negatively charged sulfonate groups of SCX4 strongly interact with the positively charged amine groups in DPPS and DPPE, as indicated by intense radial distribution functions between SCX4 sulfur atoms and nitrogen atoms of DPPE and DPPS. Unexpectedly, the sulfonate group of SCX4 are also close to the phosphate groups of DPPE or DPPS, though they are both negatively charged which could lead to repulsive interactions. However, there is the screening effect from Na^+^ counterions associated with DPPS to allow phosphate groups to approach the sulfonate groups in SCX4. Furthermore, water molecules in the aqueous subphase promote interactions between sulfonate groups and lipid headgroups, increasing proximity to phosphorus atoms. SCX4 does not exhibit affinity for DPPC or DOPC in CM models despite these lipids also having positively charged amine groups. This lack of interaction may be due to the choline headgroup of DPPC, which contains fewer hydrogen-bond donors than ethanolamine in DPPE and serine in DPPS,^91^ reducing the potential for interaction. SCX4 has no affinity for cholesterol in the CM models, most likely due to its preference for the polar headgroup environment over the hydrophobic regions around cholesterol. In the HM models, the majority of SCX4 molecules exhibit a preference for residing within the subphase, with only a minority interacting with cholesterol, which is located near the lipid tails. In contrast to the CM models, SCX4 is likely to migrate toward the hydrophobic regions near cholesterol in the absence of DPPS, driven by van der Waals forces and potential hydrophobic interactions. In summary, MD simulations indicated that SCX4 molecules exhibit a higher preference for interacting with CM models, probably due to the close proximity of SCX4 molecules to the interface. The presence of DPPS appears to facilitate SCX4 approach to the air–water interface. Conversely, SCX4 exhibits even fewer interactions with HM models. Among those that do interact, they tend to localize closer to the lipid tails, possibly influenced by cholesterol’s attraction to these regions.

**Figure 6:**
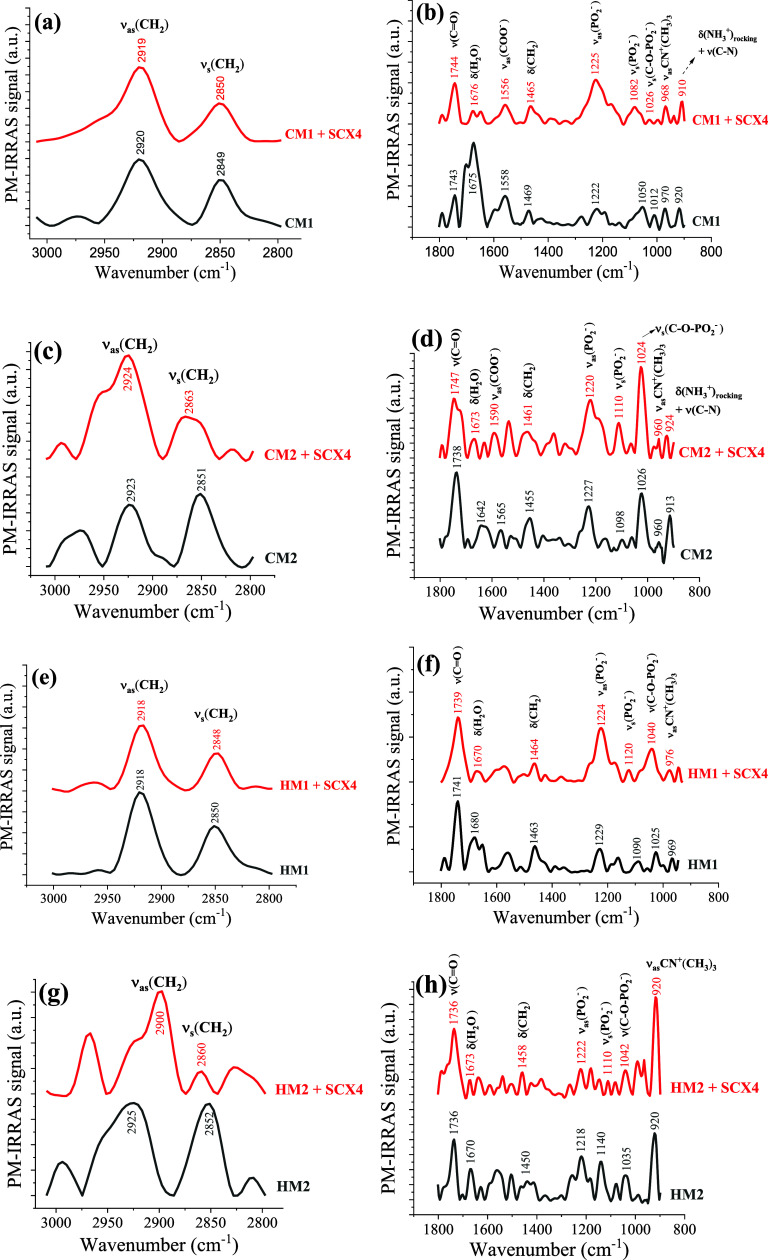
PM-IRRAS spectra for the monolayers of CM1 (a, b), CM2 (c, d), HM1 (e, f), and HM2 (g, h) models in the absence and presence of 30 μM SCX4 at π = 30 mN/m, in the range of 3000–2800 and 1800–900 cm^–1^.

The PM-IRRAS technique was employed to investigate the molecular-level interactions between SCX4 and the membrane models. The spectra for CM1, CM2, HM1, and HM2 monolayers on PBS subphase, with and without 30 μM of SCX4, are shown in Figure 6. The left panel pertains to alkyl chains (3000–2800 cm^–1^), while the right panel corresponds to the headgroups (1800–900 cm^–1^). The band assignments are given in Table 2. SCX4 interacts predominantly with the polar groups of CM1 and HM1, leading to changes in vibrational modes associated exclusively with the headgroups (Figure 6b,f). Although no significant changes were observed in the hydrophobic region, a decrease in the *I*_s_/*I*_as_ ratio was noted, indicating an increase in the conformational order of lipid alkyl chains^92^ induced by SCX4 (Figure 6a,e). Specifically, the incorporation of SCX4 into CM1 and HM1 monolayers decreased *I*_s_/*I*_as_ ratios from 0.69 to 0.50, and from 0.59 to 0.56, respectively.

**Table 2:** Vibrational Assignments of the Main Bands for the Cancer (CM1 and CM2) and Healthy Membrane (HM1 and HM2) Langmuir Monolayers along with the Shifts Induced by SCX4 Incorporation

	CM1 model (cm^–1^)		HM1 model (cm^–1^)		CM2 model (cm^–1^)		HM2 model (cm^–1^)	
assignment	on PBS	on SCX4	on PBS	on SCX4	on PBS	on SCX4	on PBS	on SCX4
ν_as_(CH_2_)	2920	2919	2918	2918	2923	2924	2925	2900
ν_s_(CH_2_)	2849	2850	2850	2848	2851	2863	2852	2860
ν(C = O)	1743	1744	1741	1739	1738	1747	1736	1736
δ(H_2_O)	1675	1676	1680	1670	1642	1673	1670	1673
ν_as_(COO^–^)	1558	1556	–	–	1565	1590	–	–
δ(CH_2_)	1469	1465	1463	1464	1455	1461	1450	1458
ν_as_(PO_2_^–^)	1222	1225	1229	1224	1227	1220	1218	1222
ν_s_(PO_2_^–^)	1050	1082	1090	1120	1098	1110	1140	1110
ν(C–O–PO_2_^–^)	1012	1026	1025	1040	1026	1024	1035	1042
ν_as_(CN^+^(CH_3_)_3_	970	968	969	976	960	960	920	920
δ(NH_3_^+^)_rocking_ + ν(C–N)	920	910	–	–	913	924	–	–

**Figure 7:**
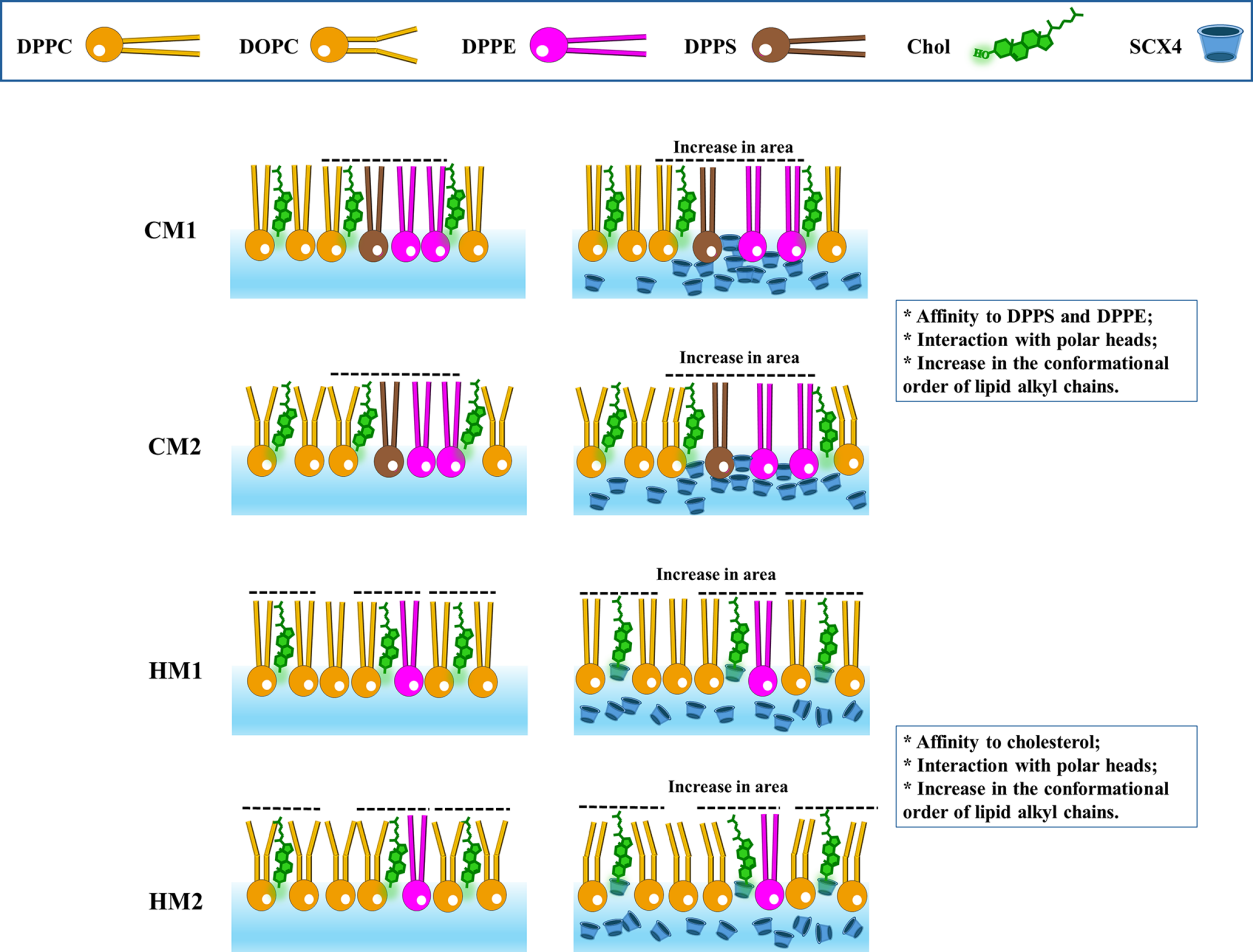
Scheme illustrating the interactions between SCX4 and membrane models at the air–water interface.

The scheme in Figure 7 illustrates how SCX4 molecules interact with the polar headgroups, resulting in an ordering of lipid tails and an increase in the molecular area, as indicated by the surface pressure–area isotherms shown above. The bending mode of water (δH2O) at around 1675 cm^–1^ decreased in intensity upon SCX4 interaction, suggesting complete coverage of the air/water interface by the monolayers. Upon interaction with SCX4, shifts to higher wavenumbers were observed in both CM1 and HM1 models for ν_s_(PO_2_^–^) bands, from 1050 to 1082 cm^–1^ and from 1090 to 1120 cm^–1^, and for ν(C–O–PO_2_^–^) bands, from 1012 to 1026 cm^–1^ and from 1025 to 1040 cm^–1^, respectively. These blueshifts arise from interactions with the lipid phosphate groups and suggest dehydration of the phosphate group.^93,94^ The band related to ν_as_(PO_2_^–^) shifted from 1222 to 1225 cm^–1^ for the CM1 model and from 1229 to 1224 cm^–1^ for the HM1 model in the presence of SCX4. For the CM1 model, simulations indicated a significant affinity between SCX4 molecules and DPPS and DPPE phospholipids, which can explain the ∼250% increase in PM-IRRAS intensity of the phosphate band. Although simulations suggest that the sulfur atom of SCX4 remains predominantly distant from the nitrogen and phosphorus atoms of DPPC and DPPE phospholipids in HM1 and that most SCX4 molecules reside within the subphase, this interaction is sufficient to alter the intensity of phosphate bands in PM-IRRAS spectra.

In the models containing the unsaturated phospholipid DOPC (HM2 and CM2), the calixarene derivative affects the lipid tail organization, as demonstrated by the displacement of bands associated with the hydrophobic tails. The ν_as_(CH_2_) band of the hydrophobic tails shifts from 2923 to 2924 cm^–1^ and from 2925 to 2900 cm^–1^ upon incorporation of SCX4 into the CM2 and HM2 models, respectively, while the ν_s_(CH_2_) band shifts from 2851 to 2863 cm^–1^ and from 2852 to 2860 cm^–1^, respectively. The incorporation of SCX4 into CM2 and HM2 monolayers also changed the I_s_/I_as_ ratios from 1.16 to 0.42, and from 0.99 to 0.22, respectively. These significant shifts may be related to the presence of the unsaturated lipid, DOPC, in the membrane models. For the increased fluidity and disorder in unsaturated lipid membranes provide more accessible sites for SCX4, leading to more pronounced changes in the vibrational modes of the lipid tails. The increase in the conformational order of lipid tails is depicted in Figure 7, where the interaction of SCX4 with polar headgroups leads to a reorganization of the lipid tails, indicating that the calixarene can affect the lipid tails without direct interaction. In contrast, saturated lipids, with their more rigid and tightly packed structure, provide fewer interaction opportunities for calixarenes, leading to less significant shifts in the hydrophobic region of the PM-IRRAS spectra. The shifts induced by SCX4 on the headgroups spectra for CM2 and HM2 in Figure 6d,h are similar to those on CM1 and HM1 models. Shifts to higher wavenumbers occurred in ν_s_(PO_2_^–^) bands, from 1098 to 1110 cm^–1^ and 1140 to 1110 cm^–1^, respectively. Also noted were shifts for ν(C–O–PO_2_^–^) bands from 1026 to 1024 cm^–1^ and 1035 to 1042 cm^–1^, respectively. The band assigned to ν_as_(PO_2_^–^) shifted from 1227 to 1220 cm^–1^ for the CM2 model and from 1218 to 1222 cm^–1^ for the HM2 model in the presence of SCX4. Dynamic molecular simulations suggested that the SCX4 derivative primarily interacts with the polar heads of the membrane models. Therefore, in HM2 and CM2, the interaction with the polar head groups likely influenced the electrostatic and van der Waals forces acting on the lipid tails, thereby inducing changes in the overall membrane structure and altering the conformational order of the lipid tails.

Considering the multiple PM-IRRAS spectra for comparison and the diverse effects of SCX4 on the membrane models, we employed the multidimensional projection technique IDMAP to analyze the effects from SCX4. The primary advantage of IDMAP is its ability to compare entire spectral regions and interpret all SCX4-induced changes, rather than focusing solely on specific bands. This approach was chosen because analyzing individual bands does not reveal a straightforward relationship, as indicated in IDMAP plots in Figure S10. For example, while SCX4 affects the headgroups of CM1 and HM1 more than the lipid tails, there is no consistent pattern of which membrane model is more impacted overall. A similar variability is observed in CM2 and HM2, where SCX4 impacts both membrane regions to varying degrees across different bands. In the hydrophobic lipid tails region (Figure S10a), large effects were observed in CM2 and HM2, consistent with the observed larger shifts in this region. When comparing healthy and cancerous membranes, SCX4 effects were more pronounced on HM1 and HM2 compared to CM1 and CM2. This observation may be attributed by SCX4’s affinity for cholesterol in healthy models, given that cholesterol resides near lipid tails. In contrast, the headgroups region (Figure S10b) shows a higher impact of SCX4 on CM1 and HM1 than on CM2 and HM2 that contain unsaturated lipids. In comparing cancer and healthy models, SCX4 affects CM1 and CM2 to a larger extent than it does to HM1 and HM2. The main conclusion is that SCX4 effects are highly dependent on membrane composition.

Since the effects of SCX4 depend on membrane composition, future research should focus on more realistic models by incorporating membrane proteins, particularly those related to transport carriers, to further explore their interactions with SCX4. Additionally, since Langmuir monolayers represent only one leaflet of the cell membrane and have limitations in studying transmembrane processes, we plan to investigate bilayer membrane models. These studies will provide further understanding of SCX4 interactions and their impact on membrane dynamics. Furthermore, based on our conclusion that SCX4 molecules do not disrupt the overall membrane structure, we plan to conduct concentration-dependent cytotoxicity assessments to evaluate SCX4’s impact on cellular health and viability. These future studies will expand our understanding of SCX4’s potential as a drug carrier, particularly in drug delivery systems, while further advancing the molecular insights gained from this work.

## Conclusions

We investigated the interactions between the *p*-sulfonic acid calix[4]arene derivative (SCX4) and Langmuir monolayers mimicking the outer leaflet of plasma membranes of healthy (HM1 and HM2) and cancerous (CM1 and CM2) cells. Through surface pressure isotherms, surface compressional moduli, Brewster angle microscopy, and PM-IRRAS spectroscopy combined with molecular dynamics (MD) simulations, we elucidated the molecular-level mechanisms of SCX4-membrane interactions. Our results show that SCX4 molecules interact differently with healthy and cancerous membrane models, inducing changes in monolayer packing. PM-IRRAS data indicated that SCX4 preferentially interacts with lipid headgroups, particularly in saturated lipid models (CM1 and HM1). In models containing unsaturated lipids (CM2 and HM2), headgroups were affected significantly, with shifts also observed in hydrophobic tails due to the increased fluidity and sensitivity of unsaturated lipids to interfacial changes. MD simulations supported PM-IRRAS findings, showing that SCX4 molecules remain close to lipid headgroups for the cancer membrane models and do not migrate to the tails of lipids due to their electrostatic interactions with DPPS and DPPE molecules. Additionally, MD simulations revealed that SCX4 exhibits enhanced affinity for cholesterol-rich domains in healthy membranes. The overall results suggest that the interactions of SCX4 with membrane headgroups may contribute to its biocompatibility, thereby implying the maintenance of the overall membrane structure. Moreover, the entry mechanism of sulfonatocalixarene derivatives into cells likely involves external stimuli such as pH, heat, or proteins, rather than passive incorporation into the membrane. Our study enhances the understanding of calixarene interactions with cell membranes and features the importance of studying lipid membranes alongside other drug carriers. It provides molecular-level insight into SCX4 interactions with cell membranes, emphasizing the roles of cholesterol and DPPS. Furthermore, it determines the localization of SCX4 within the membrane model. This type of knowledge could aid in developing drug carriers that specifically interact with plasma membranes without damaging their structure, facilitating efficient cellular entry.

## Supporting Information Available

Surface pressure–area isotherms and compressional moduli for pure monolayers of DPPC, DOPC, DPPE, DPPS, and cholesterol on a PBS subphase; surface pressure–area isotherms, compressional moduli, and BAM images for DPPC, DOPC, DPPE, DPPS, and cholesterol monolayers on PBS with varying SCX4 concentrations; IDMAP plots for π–*A* isotherm data; table of parameters for DPPC, DOPC, DPPE, DPPS, and cholesterol monolayers on PBS with SCX4; PM-IRRAS spectra for DPPC, DOPC, DPPE, DPPS, and cholesterol monolayers with and without SCX4; IDMAP plots for π–*A* isotherm and compressional modulus data for membrane models with SCX4; volumetric density profiles for HM2 and CM2 monolayers; density profiles for (water + SCX4)–(HM1) interface; radial distribution functions for atom distances in membrane models and SCX4; PM-IRRAS data analysis using IDMAP (PDF)
