# Supplementary material for: Nanoarchitectonics and Simulation on the Molecular-Level Interactions between p-Sulfonic Acid Calix[4]arene and Langmuir Monolayers Representing Healthy and Cancerous Cell Membranes
Source: Langmuir. 2024 Dec 12;40(51):27010–27. doi: 10.1021/acs.langmuir.4c03948 (PMC11673576; doi:10.1021/acs.langmuir.4c03948)
Supplement: Supplementary file 1 — la4c03948_si_001.pdf [file la4c03948_si_001.pdf]

Supporting Information:

Nanoarchitectonics and simulation on the molecular-level interactions between *p*-sulfonic acid calix[4]arene and Langmuir monolayers representing healthy and cancerous cell membranes

Ellen C. Wrobel,[1]* Lucas Stori de Lara,[2] Ângelo de Fátima,[3] Osvaldo N. Oliveira Jr.[1]*

[1]*São Carlos Institute of Physics, University of São Paulo, CP 369, São Carlos, São Paulo (SP) 13560-970, Brazil*

[2]*Department of Physics, State University of Ponta Grossa, Ponta Grossa, PR, 84030-900, Brazil.*

[3]*Department of Chemistry, Institute of Exact Sciences, Federal University of Minas Gerais, 31270-901 Belo Horizonte, MG, Brazil.*

CONTENTS

1. Langmuir monolayers of DPPC, DOPC, DPPE, DPPS, and Cholesterol (Figure S1);
2. Effect of *p*-sulfonic acid calix[4]arene on pure lipids and cholesterol monolayers (Figure S2 and S3, and Table S1);
3. Interaction of SCX4 with cancer and healthy membrane models - IDMAP analysis (Figure S4 and S5);
4. Analysis of SCX4 interactions with healthy and cancer membrane models using molecular dynamics (MD) simulations (Figures S6-S9);
5. Analysis of PM-IRRAS spectra for the healthy and cancer membrane models in the presence and absence of SCX4 (Figure S10).

*Corresponding authors:

e-mail addresses: wrobel.ellen@gmail.com (E. C. Wrobel), chu@ifsc.usp.br (O. N. Oliveira Jr.)

1. *Langmuir monolayers of DPPC, DOPC, DPPE, DPPS, and Cholesterol*

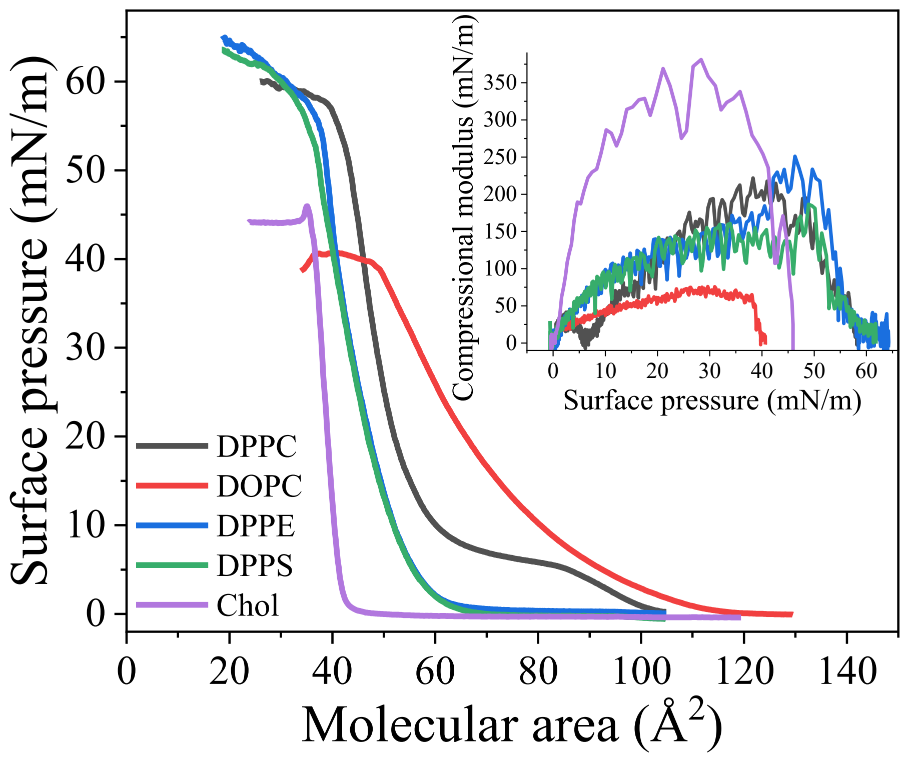

Fig. S1. Surface pressure-area (π-A) isotherms and compressional moduli (C_S^{-1}) for pure DPPC, DOPC, DPPE, DPPS, and cholesterol monolayers on PBS subphase (pH = 7.4).

2. *Effect of p-sulfonic acid calix[4]arene on pure lipids and cholesterol monolayers*

Previously, as detailed in the main manuscript, it was noted that the interaction between SCX4 and the proposed cancer and healthy membrane models depends on their composition. Therefore, we studied the effect of the calixarene derivative on the Langmuir monolayers of the membrane model components: DPPC, DOPC, DPPE, DPPS, and cholesterol. The impact of different concentrations of SCX4 (1, 10, and 30 μM) on these components is illustrated in Fig. S2, with the main parameters detailed in Table S1. Phosphatidylcholine (PC), a zwitterionic lipid, is the most abundant phospholipid in the outer leaflet of healthy membranes [1–3]. SCX4 expands the DPPC isotherm (Fig. S2a) at large areas but does not significantly affect the liquid-condensed phase, likely indicating that the calixarene remains in the subphase, interacting only with the polar headgroups of DPPC. The monolayer expansion at low pressures may result from weak intermolecular forces between the sulfonate groups of SCX4 and the headgroups of DPPC. The presence of SCX4 decreases the surface compressional modulus, suggesting that SCX4 alters the mechanical properties and makes the DPPC monolayer more compressible. BAM images show that SCX4 has little effect on the morphology of DPPC monolayer. The chiral multilobed liquid-condensed (LC) domains enclosed by the liquid-expanded (LE) phase in the pure DPPC monolayer are replaced by smaller, rounded domains in SCX4-containing monolayers. Additionally, SCX4 does not affect the morphology of the DPPC monolayer in the condensed phase, indicating that the major interactions between DPPC and SCX4 occur at lower pressures. For the DOPC monolayer, it was found that SCX4 increases the molecular area without changing the compressional modulus of DOPC (Fig. S2b). These results suggest that monolayer fluidity is crucial for how SCX4 interacts with cell membranes, with unsaturated lipids likely playing a key role in the transport of calixarenes through the membrane. Brewster angle microscopy (BAM) images indicated that SCX4 has no effect on the morphology of DOPC. This finding suggests that SCX4's biocompatibility is due to its interaction with phosphatidylcholine lipids without disrupting their structure.

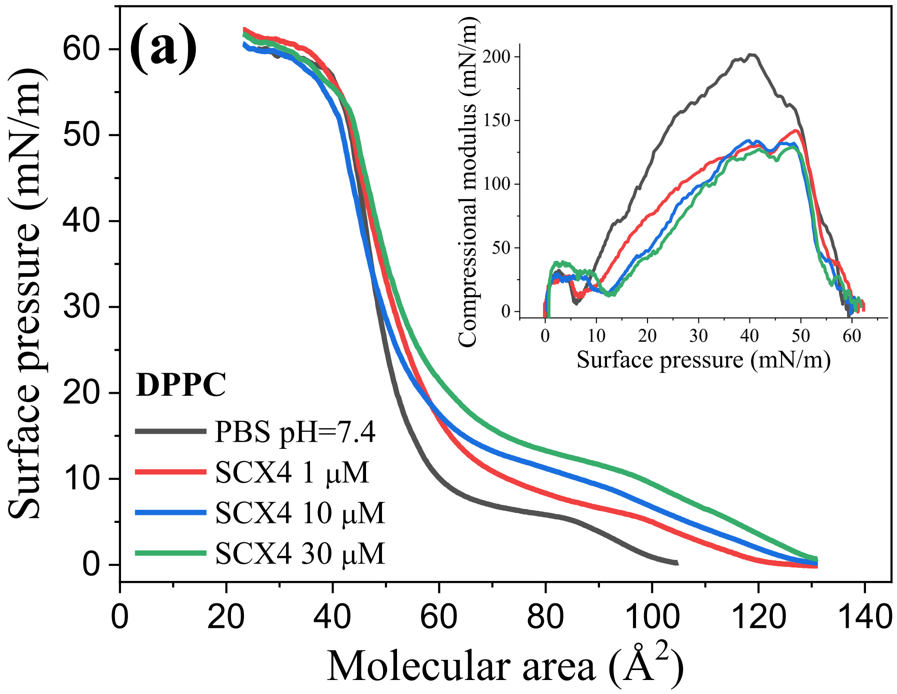

(a)
Surface pressure (mN/m)
Molecular area (Å2)
DPPC
PBS pH=7.4
SCX4 1 μM
SCX4 10 μM
SCX4 30 μM
Compressional modulus (mN/m)
Surface pressure (mN/m)

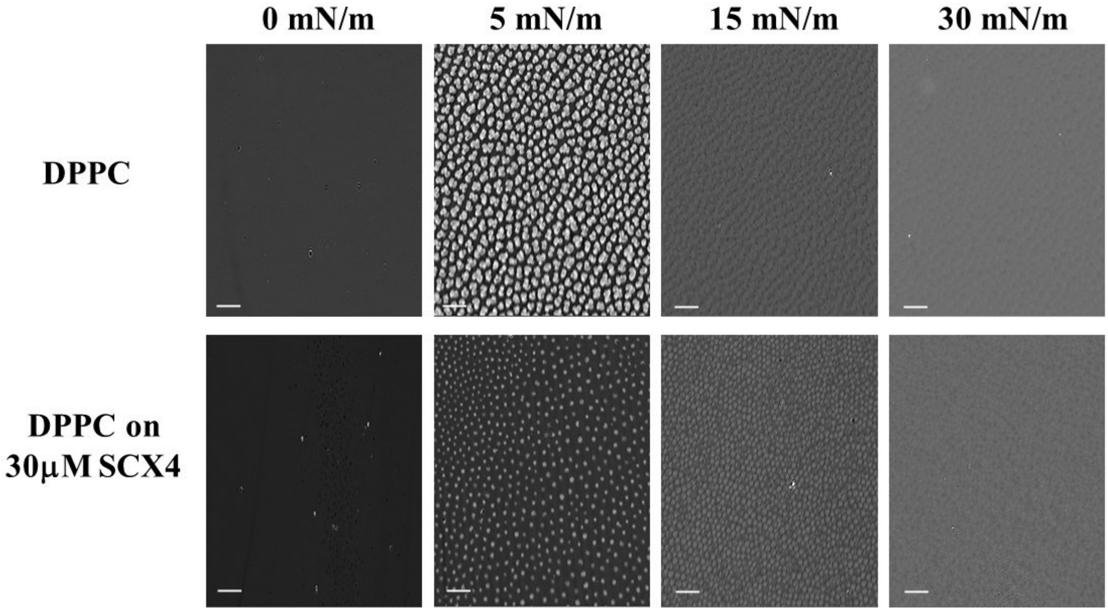

0 mN/m
5 mN/m
15 mN/m
30 mN/m
DPPC
DPPC on 30μM SCX4

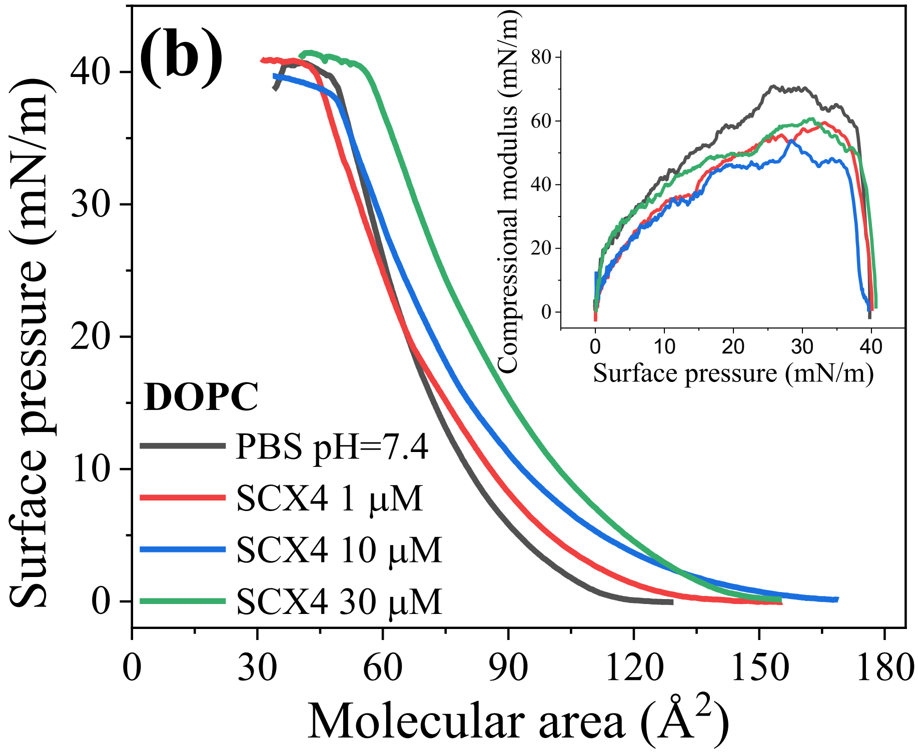
(b)
DOPC
PBS pH=7.4
SCX4 1 μM
SCX4 10 μM
SCX4 30 μM
Surface pressure (mN/m)
Molecular area (Å2)
Compressional modulus (mN/m)
Surface pressure (mN/m)

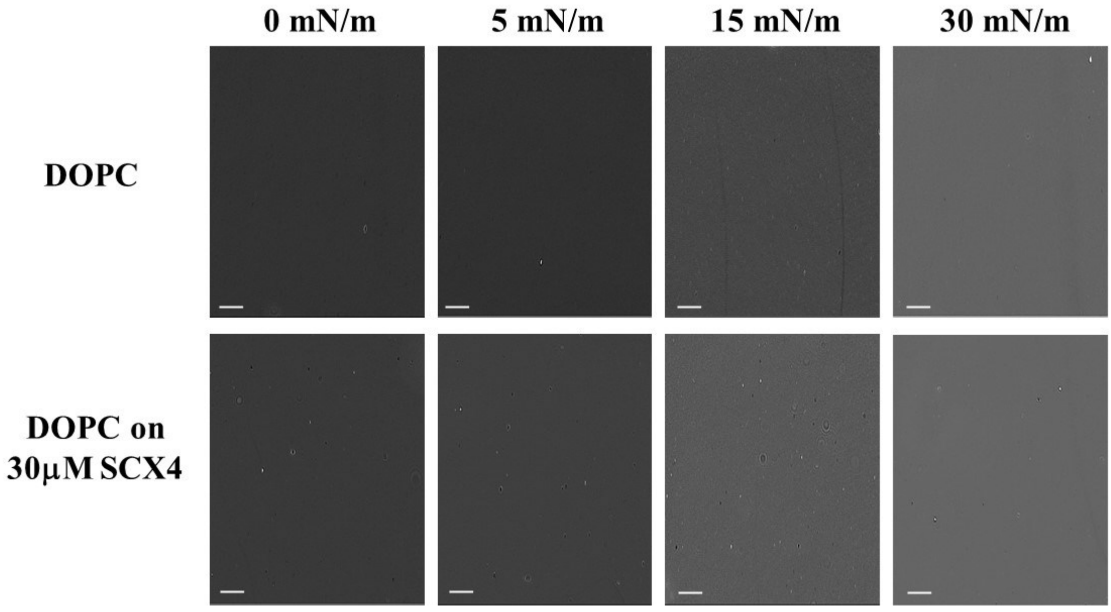
0 mN/m
5 mN/m
15 mN/m
30 mN/m
DOPC
DOPC on 30μM SCX4

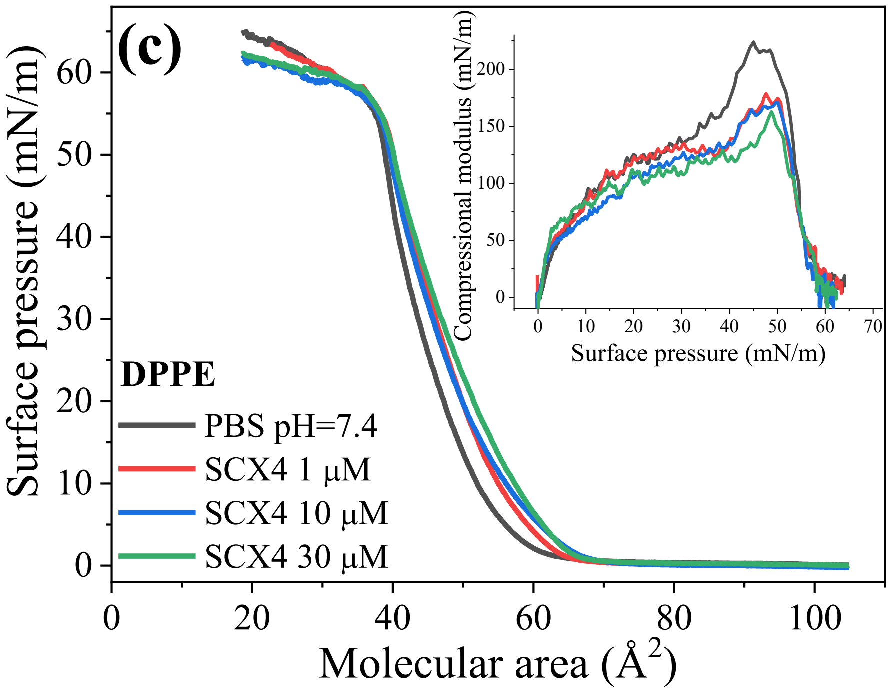
(c)
Surface pressure (mN/m)
Molecular area (Å^2)
0
20
40
60
80
100
0
10
20
30
40
50
60
DPPE
PBS pH=7.4
SCX4 1 μM
SCX4 10 μM
SCX4 30 μM
Compressional modulus (mN/m)
Surface pressure (mN/m)
0
50
100
150
200
0
10
20
30
40
50
60
70

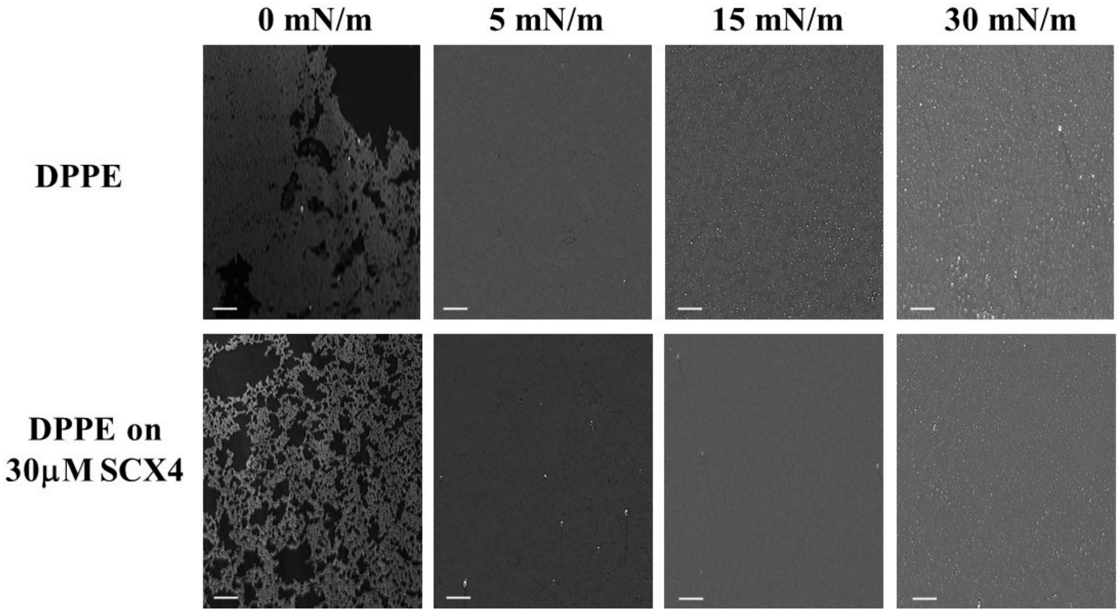
0 mN/m
5 mN/m
15 mN/m
30 mN/m
DPPE
DPPE on
30μM SCX4

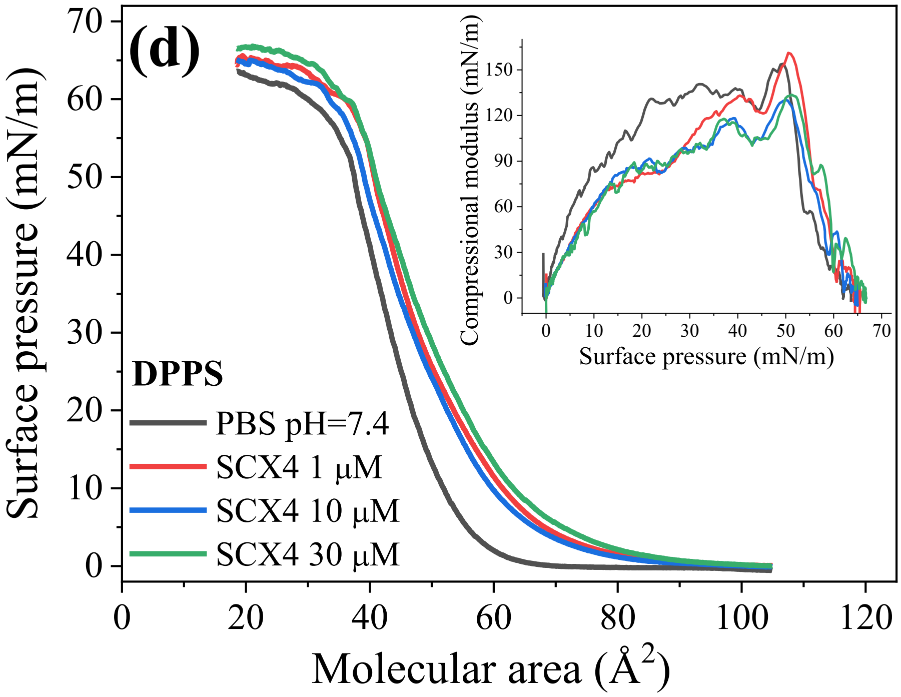
(d)
Surface pressure (mN/m)
Molecular area (Å^2)
DPPS
PBS pH=7.4
SCX4 1 μM
SCX4 10 μM
SCX4 30 μM
Compressional modulus (mN/m)
Surface pressure (mN/m)

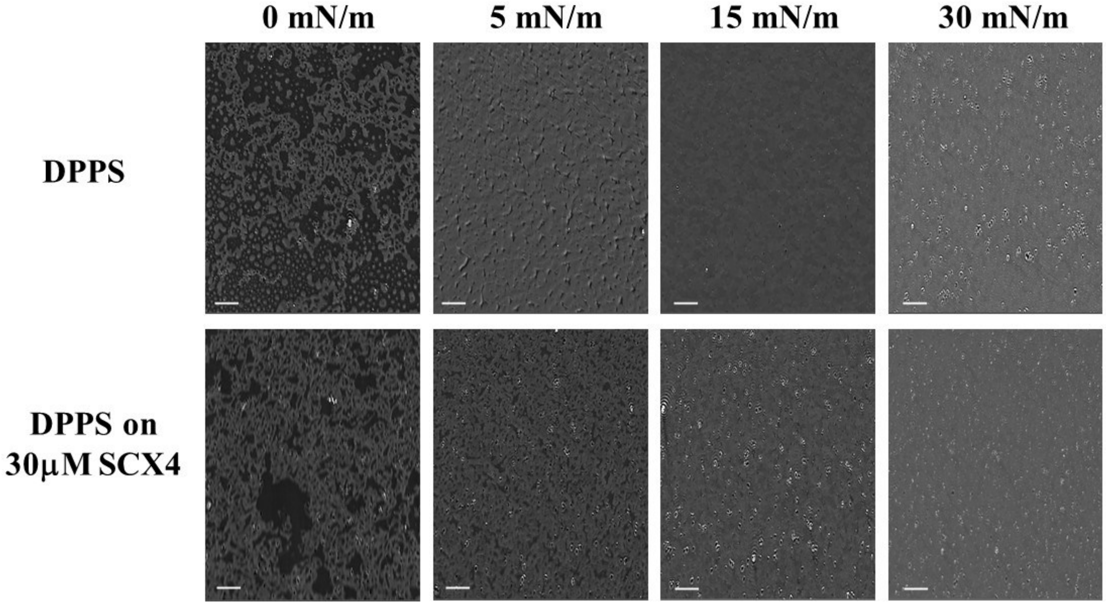
0 mN/m
5 mN/m
15 mN/m
30 mN/m
DPPS
DPPS on 30μM SCX4

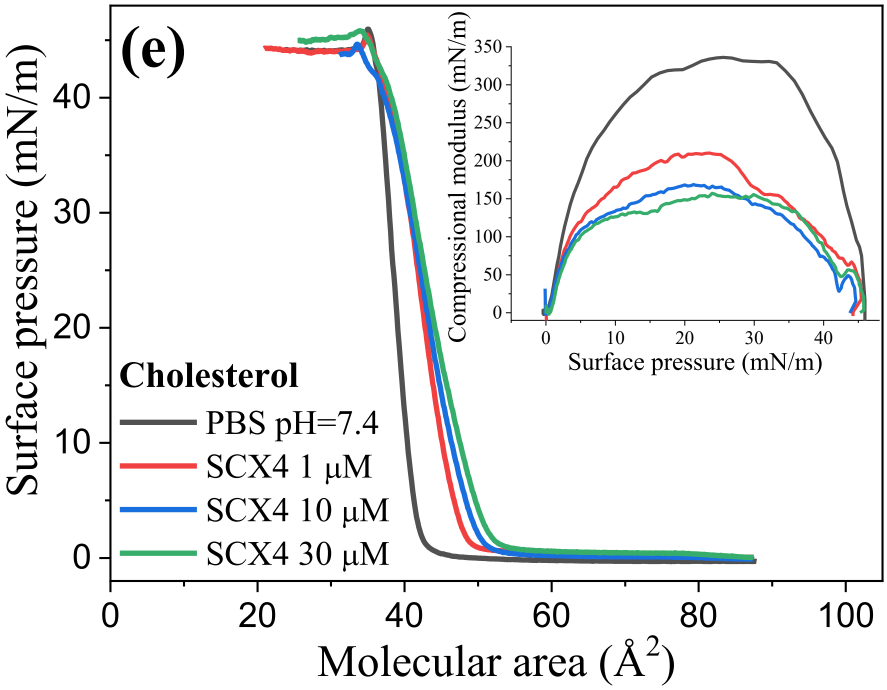

(e)
Surface pressure (mN/m)
Molecular area (Å^2)
Cholesterol
PBS pH=7.4
SCX4 1 μM
SCX4 10 μM
SCX4 30 μM
Compressional modulus (mN/m)
Surface pressure (mN/m)

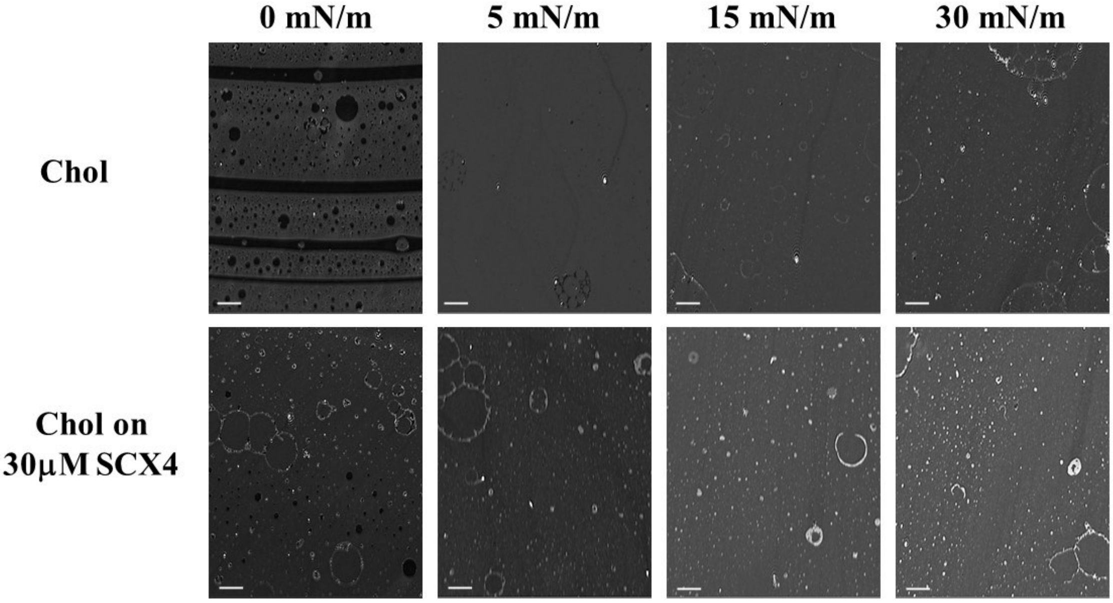

0 mN/m
5 mN/m
15 mN/m
30 mN/m
Chol
Chol on 30μM SCX4

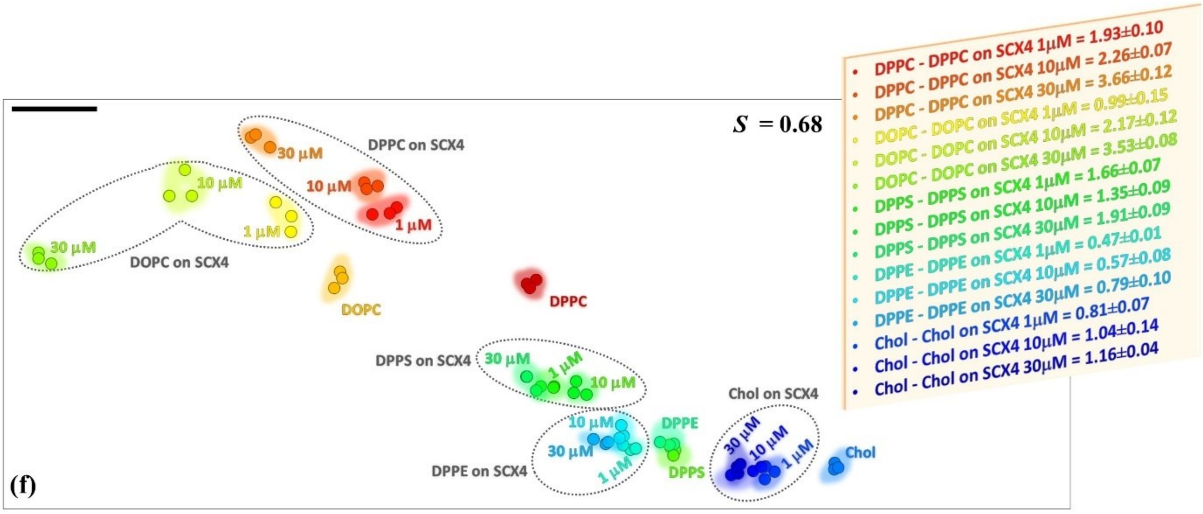

Fig. S2. Surface pressure-area (π-A) isotherms, compressional moduli (C_S^{-1}), and BAM images (the scale bar represents 50 μm) for monolayers of (a) DPPC, (b) DOPC, (c) DPPE, (d) DPPS, and (e) Chol on PBS subphase containing 0, 1, 10, and 30 μM of SCX4. IDMAP plots (f) for the referred π-A isotherms data at the pressure range of 0 – 35 mN/m. Axes are intentionally not labeled, as the IDMAP plot emphasizes the relative distances between data points. The black bar is included as a reference guide.

Table S1. Parameters for DPPC, DOPC, DPPE, DPPS, and Chol monolayers on PBS subphase containing 1, 10, and 30 μM of SCX4, where **Aex**: extrapolated area, $\mathbf{A_{30}}$: molecular area at 30 mN/m, π_{col}: collapse pressure, $\mathbf{Cs^{-1}_{max}}$: maximum compressional modulus, and $\mathbf{Cs^{-1}_{30}}$: compressional modulus at 30 mN/m.

	A_{ex} (Å^2)	A_{30} (Å^2)	π_{col} (mN/m)	Cs^{-1}_{max} (mN/m)	Cs^{-1}_{30} (mN/m)
DPPC on PBS	57.4±0.7	48.3±0.3	55.8±1.4	201±8	167±12
DPPC on SCX4 1μM	64.3±0.5	51.5±0.5	60.1±0.2	142±5	110±4
DPPC on SCX4 10μM	64.0±0.5	49.6±0.2	57.6±0.6	135±10	98±5
DPPC on SCX4 30μM	64.6±0.5	52.7±0.5	57.7±0.6	130±8	92±7
DOPC on PBS	83.8±0.2	56.6±0.3	40.6±0.4	70±6	69±6
DOPC on SCX4 1μM	87.8±0.3	54.4±0.2	40.8±0.5	59±4	57±4
DOPC on SCX4 10μM	98.7±0.2	58.3±0.4	38.7±0.3	53±5	50±5
DOPC on SCX4 30μM	108.6±0.4	68.1±0.3	40.8±0.4	60±6	59±8
DPPE on PBS	51.5±0.3	43.6±0.1	57.1±0.2	220±10	135±9
DPPE on SCX4 1μM	55.8±0.2	46.1±0.2	57.6±0.3	176±5	132±8
DPPE on SCX4 10μM	55.2±0.3	45.7±0.2	57.1±0.3	170±4	124±8
DPPE on SCX4 30μM	57.1±0.4	47.0±0.3	58.1±0.4	161±6	112±7
DPPS on PBS	54.6±0.5	43.4±0.4	58.8±1.5	154±9	135±7
DPPS on SCX4 1μM	59.4±0.6	47.4±0.7	60.6±0.4	161±7	105±6
DPPS on SCX4 10μM	58.7±0.3	47.0±0.6	61.7±0.3	130±8	97±10
DPPS on SCX4 30μM	61.5±0.4	49.3±0.3	63.5±0.5	133±6	95±7
Chol on PBS	41.6±0.2	37.9±0.1	45.9±0.3	340±5	330±6
Chol on SCX4 1μM	47.5±0.6	40.7±0.3	45.4±0.4	210±12	165±10
Chol on SCX4 10μM	49.6±0.1	40.8±0.2	44.6±0.5	168±5	143±7
Chol on SCX4 30μM	50.8±0.2	41.3±0.3	45.7±0.4	155±6	154±8

Phosphatidylethanolamine (PE) is found in both the outer and inner leaflets of plasma membranes, primarily in the inner leaflet; however, in many cancers, its distribution reverses, with a preference for the outer leaflet [1,2,4]. Phosphatidylserine (PS) is typically located in the inner leaflet of the cell membrane. However, in several types of cancer, the loss of lipid asymmetry results in PS being exposed on the outer leaflet [1,4]. Hence, PE and PS lipids can be considered as cancer biomarkers due to their predominant presence in the outer layer of the membrane [1,2]. The DPPE phospholipid is minimally affected by SCX4 (Fig. S2c), showing a very small increase in molecular area and slight decrease in compressional modulus. Even increasing the SCX4 concentration did not significantly enhance its interactions with DPPE. These weak interactions are consistent with the morphological observations, as SCX4 did not cause

any notable changes in the BAM images of DPPE. In contrast, the DPPS monolayer is affected by SCX4 (Fig. S2d), as SCX4-containing monolayers exhibit a greater molecular area. However, SCX4 has little effect on the compressional modulus of DPPS. The increase in molecular area may be due to repulsion interactions between the serine groups of DPPS and the sulfonate groups of SCX4, leading to an increase in area without affecting the fluidity of the monolayer. Morphological changes were observed when SCX4 was interacting with the DPPS monolayer; aggregated domains that typically appear at 30 mN/m in the pure DPPS monolayer were observed around 5 mN/m in the presence of SCX4, when the monolayer is not yet condensed. This behavior may be related to the repulsion interactions between DPPS and SCX4. Cholesterol (Chol) is the major sterol of mammalian cell membranes, crucial for maintaining structural integrity and regulating cell membrane fluidity [5]. The incorporation of SCX4 into the Chol monolayer shifts its isotherm to higher molecular areas and significantly decreases the interfacial elasticity of the monolayer (Fig. S2e). BAM images of Chol show a coexistence of gas (dark areas) and LC (bright regions) phases at low surface pressure region (liftoff area). Upon compression, the Chol monolayer quickly becomes homogeneous, with circular condensed domains appearing at higher pressures. In the presence of SCX4, the Chol monolayers exhibit a fully condensed state with circular structures present even at 0 mN/m, whereas these structures typically appear at around 15 mN/m in the pure Chol monolayer.

The effects of SCX4 on the lipids and cholesterol were confirmed by analyzing the π-A isotherms using the IDMAP multidimensional projection technique (Fig. S2f). The IDMAP analysis indicates that SCX4 interact with all monolayers, with the degree of interaction depending on the structure of the component. The silhouette coefficient (S) was 0.68, indicating a significant level of data discrimination [6]. The relative Euclidean distances in the inset of Fig. S2f between isotherms with and without SCX4 in the subphase allow for direct comparison of the induced effects. As the SCX4 concentration increases, greater cluster separation is observed, with a more pronounced effect on the DPPC and DOPC monolayers on a PBS subphase containing 30 μM of SCX4. DPPE was the least affected lipid within the studied SCX4 concentration range, which is consistent with the π-A isotherms and BAM images.

From the spectroscopic analysis of the interactions between pure monolayers and SCX4 via PM-IRRAS (Fig. S3), we observed a trend consistent with that seen in the

membrane models (Fig. 7 in the main manuscript). SCX4 molecules primarily affect the lipid headgroups, as indicated by significant spectral shifts in the range of 1800-900 cm^{-1}. The hydrophobic tails remain largely unaffected in saturated lipids, while notable changes were observed in the spectra for the unsaturated DOPC lipid. This aligns with the HM2 and CM2 models, where increased fluidity and disorder in unsaturated lipid membranes provide more accessible sites for SCX4, resulting in more pronounced changes in the vibrational modes of the lipid tails. In contrast, the saturated lipids DPPC, DPPE, and DPPS, with their rigid and tightly packed structure, offer fewer interaction opportunities for SCX4 molecules, leading to less significant shifts in the hydrophobic region of the PM-IRRAS spectra. Thus, in DOPC, interaction with the polar headgroups likely influences the electrostatic and van der Waals forces acting on the lipid tails, leading to changes in the overall membrane structure and altering the conformational order of the lipid tails.

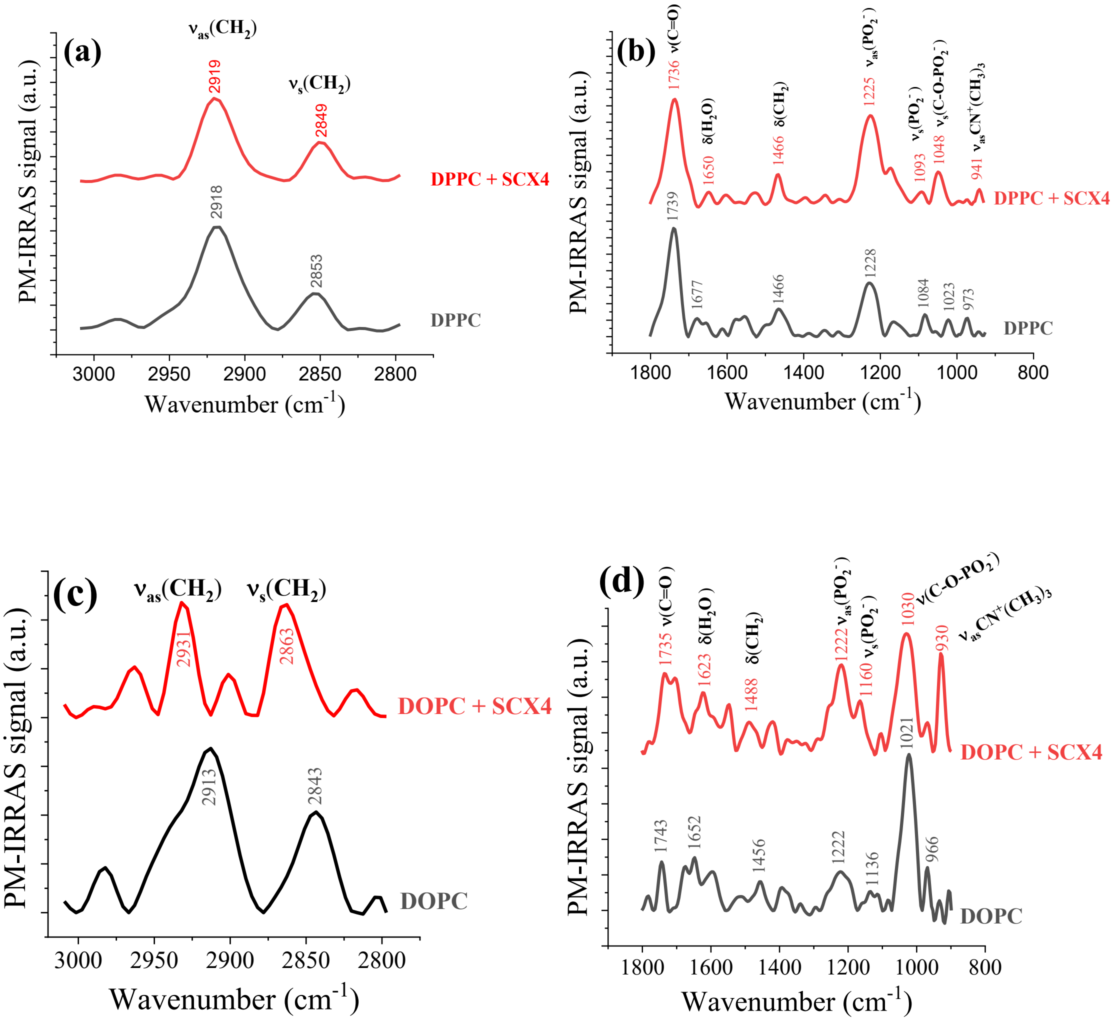

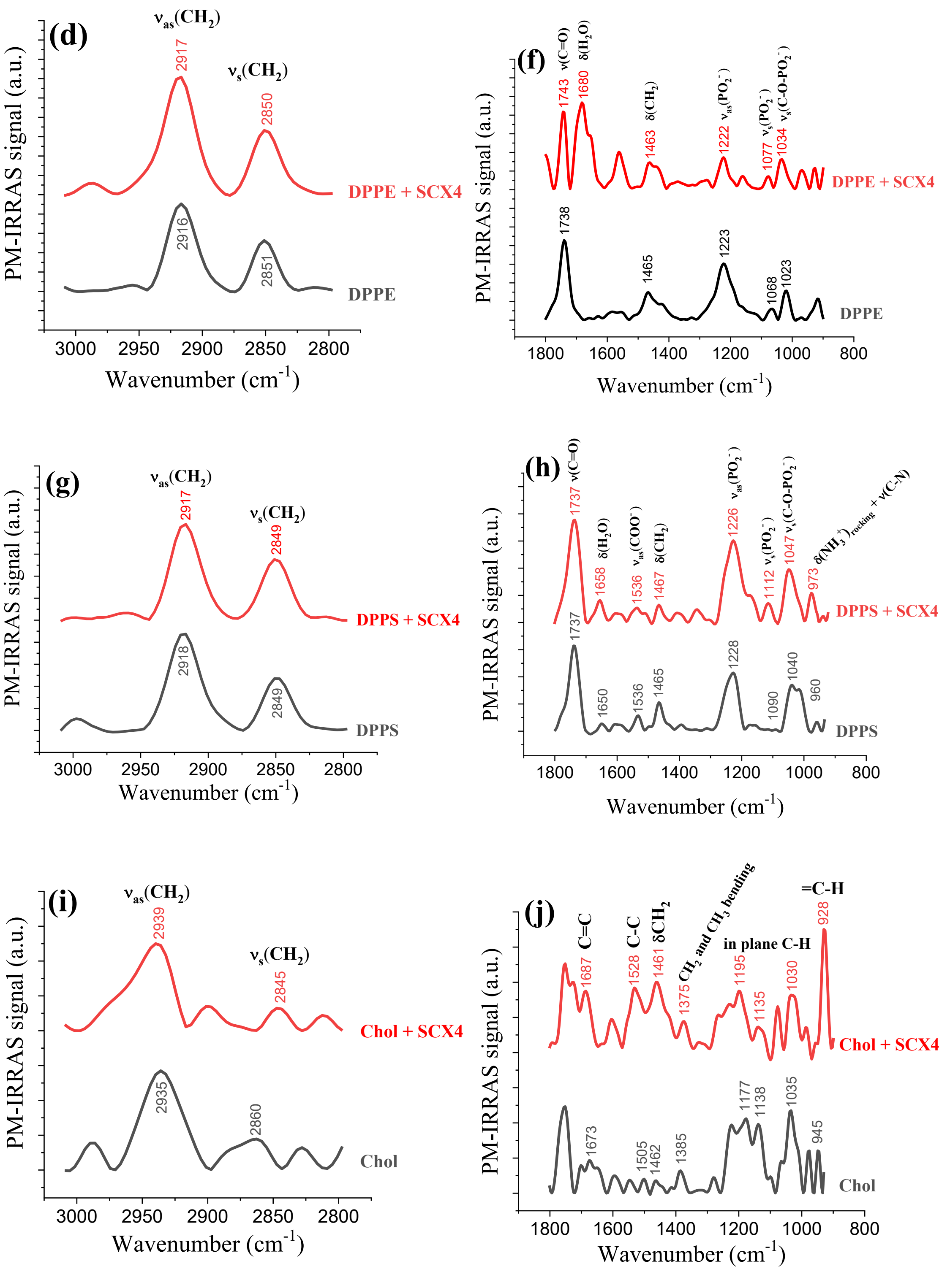

Fig. S3. PM-IRRAS spectra for the monolayers for (a, b) DPPC, (c, d) DOPC, (e, f) DPPE, (g, h) DPPS, and (i, j) Chol in the absence and presence of 30 µM SCX4 at π = 30 mN/m, in the range of 3000-2800 and 1800-900 cm^{-1}.

3. *Interaction of SCX4 with cancer and healthy membrane models - IDMAP analysis*

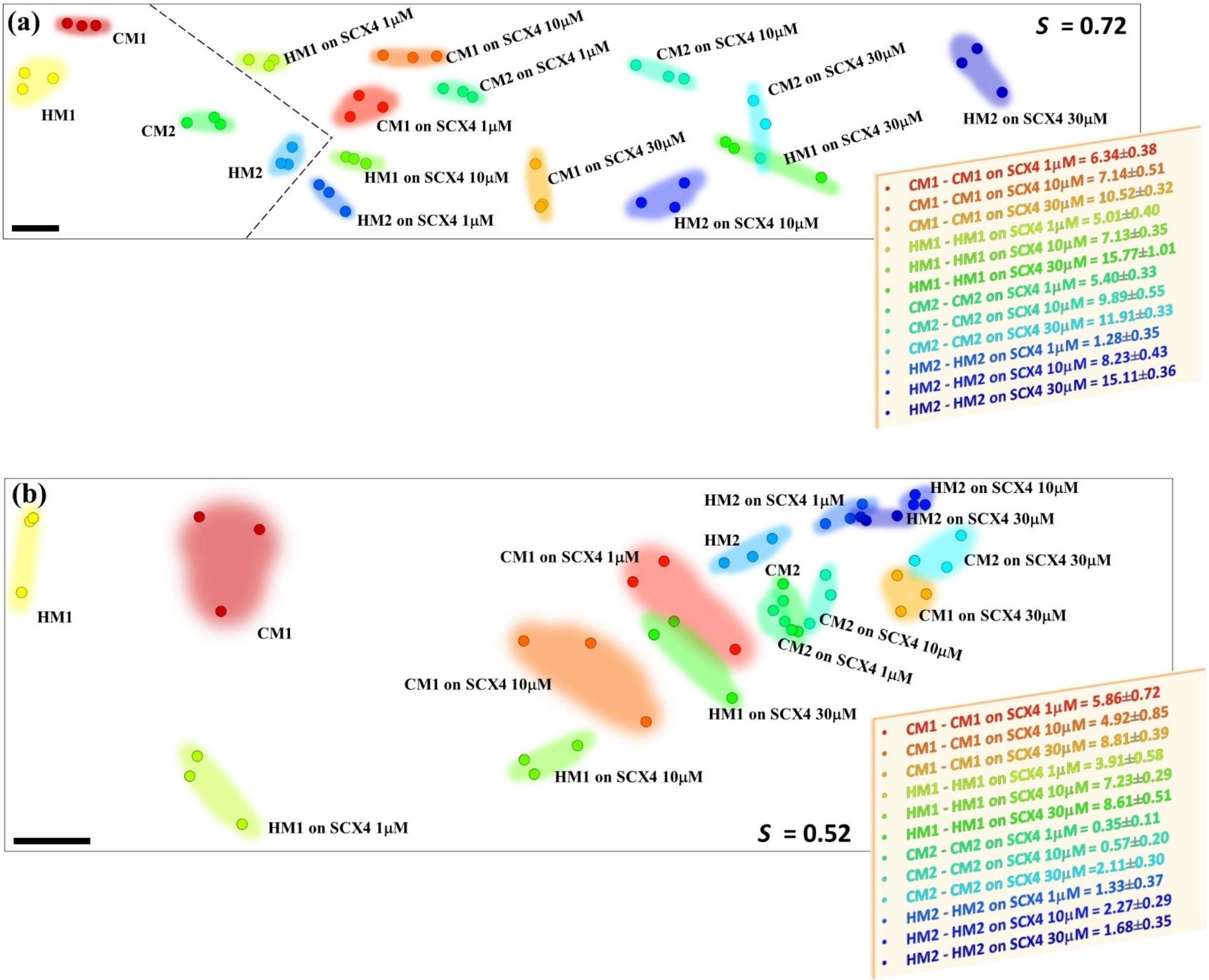

Fig. S4. IDMAP plots for (a) π-A isotherms and (b) compressional modulus data from Fig. 4 in the main manuscript, within the pressure range of 0–35 mN/m. Axes are intentionally not labeled, as the IDMAP plot emphasizes the relative distances between data points. The black bar is included as a reference guide.

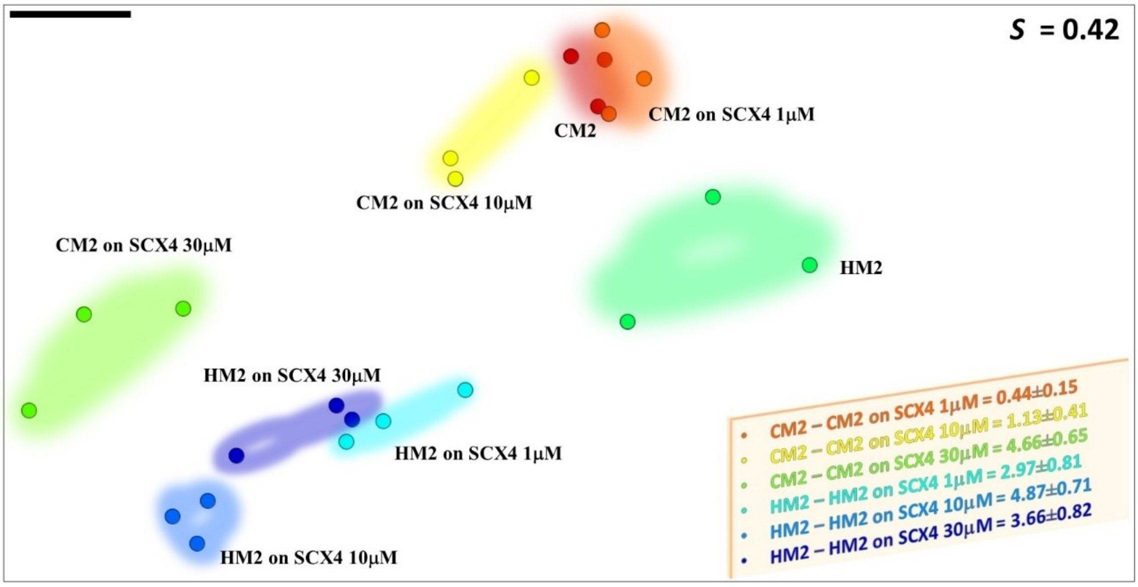

Fig. S5. IDMAP plots for the compressional modulus data in Fig. 4c and 4d of the main manuscript, at the pressure range of 0 – 35 mN/m. Axes are intentionally not labeled, as the IDMAP plot emphasizes the relative distances between data points. The black bar is included as a reference guide.

4. Analysis of SCX4 Interactions with Healthy and Cancerous Membrane Models Using Molecular Dynamics (MD) Simulations

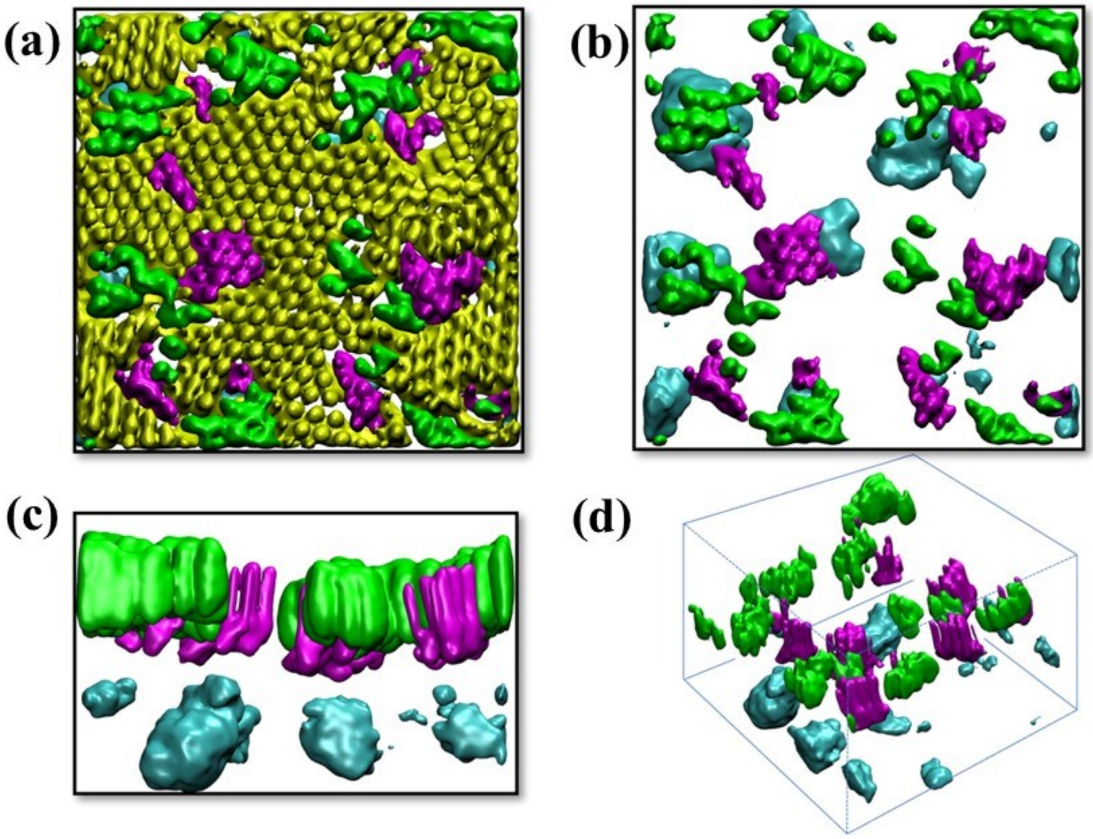

Fig. S6. (a) Top view of volumetric density profiles for HM2 monolayers at π =10 mN/m. (b) DOPC removed; (c) lateral view in (b); (d) 3D view in (b). Green, yellow, magenta, and cyan represent the cholesterol, DOPC, DPPE, and SCX4 species in the monolayer, respectively.

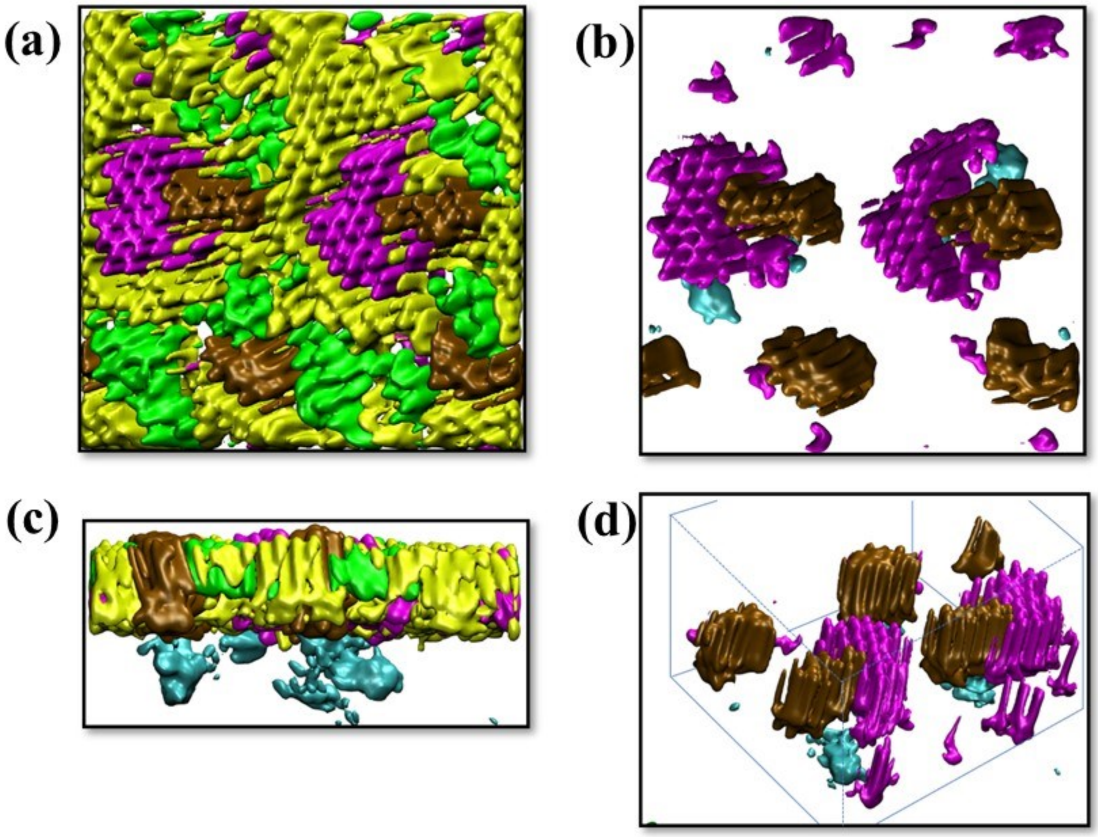

Fig. S7. (a) Top view of volumetric density profiles for CM2 monolayers at π =10 mN/m. (b) DOPC removed; (c) lateral view in (b); (d) 3D view in (b). Green, yellow, magenta, brown, and cyan represent the cholesterol, DOPC, DPPE, DPPS, and SCX4 species in the monolayer, respectively.

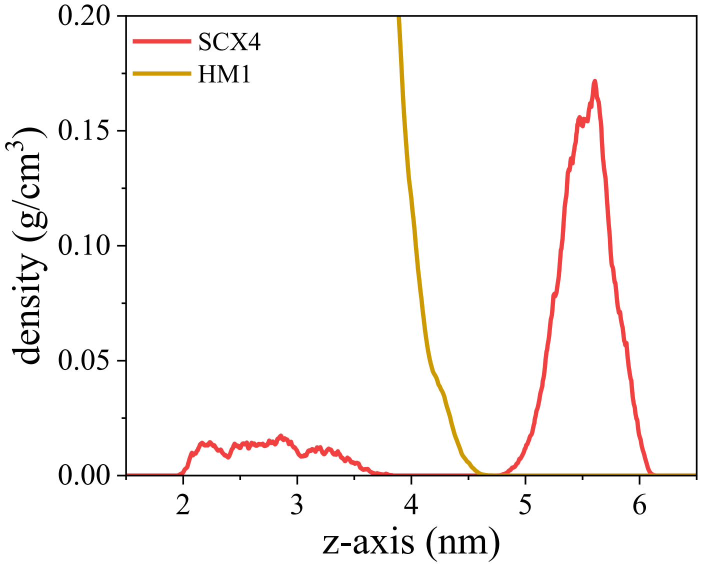

Fig. S8. Enlarged section of Fig. 6c: Density profiles for (water+SCX4)–(HM1) interface at 300K and surface pressure π = 10 mN/m.

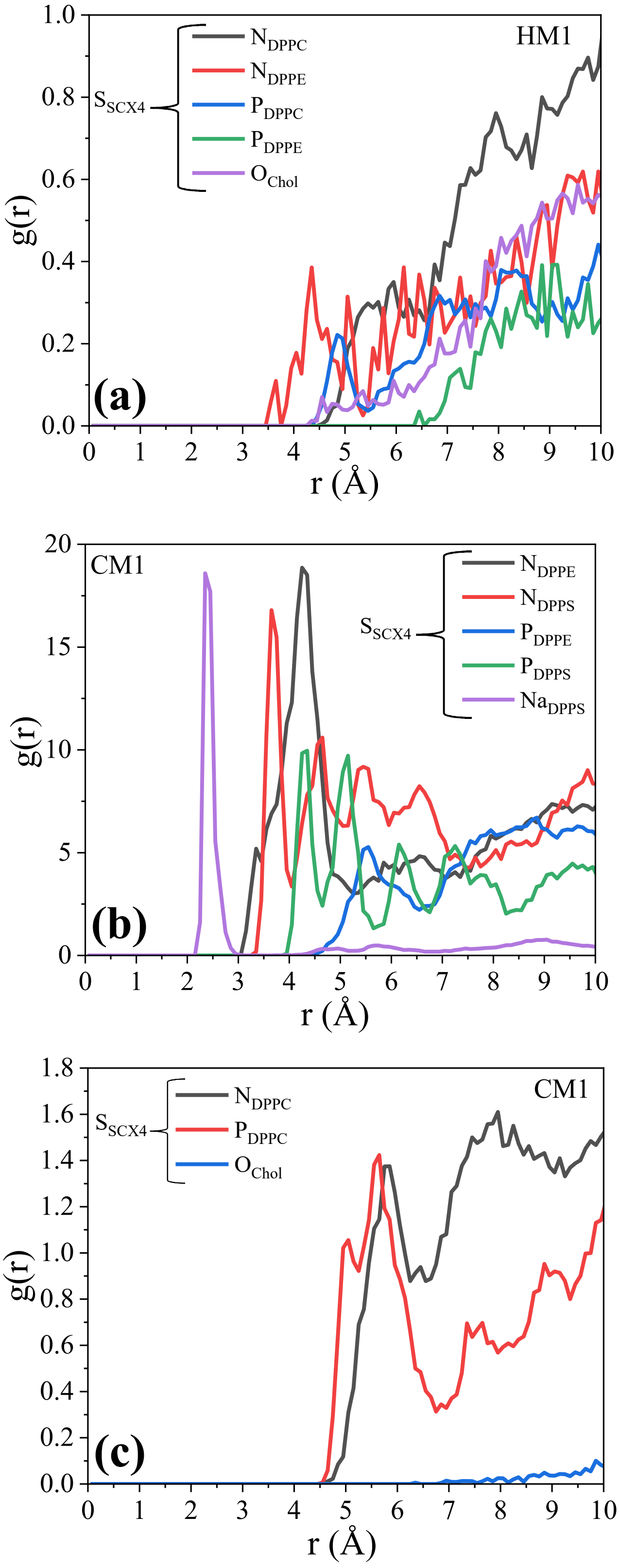

Fig. S9. Radial distribution function g(r): (a) Distance between the sulfur (S_{SCX4}) atom of SCX4 and the nitrogen and phosphorus atoms of DPPC (N_{DPPC}, P_{DPPC}) and DPPE (N_{DPPE}, P_{DPPE}), and oxygen atoms of cholesterol (O_{Chol}) in the HM1 model; (b) Distance between the sulfur (S_{SCX4}) atom of SCX4 and the nitrogen, phosphorus, and sodium atoms of DPPE (N_{DPPE}, P_{DPPE}) and DPPS (N_{DPPS}, P_{DPPS}, and Na_{DPPS}) in the CM1 model; (c) Distance between the sulfur (S_{SCX4}) atom of SCX4 and the nitrogen and phosphorus atoms of DPPC (N_{DPPC}, P_{DPPC}) and oxygen atoms of cholesterol (O_{Chol}) in the CM1 model.

5. *Analysis of PM-IRRAS spectra for the healthy and cancer membrane models in the presence and absence of SCX4*

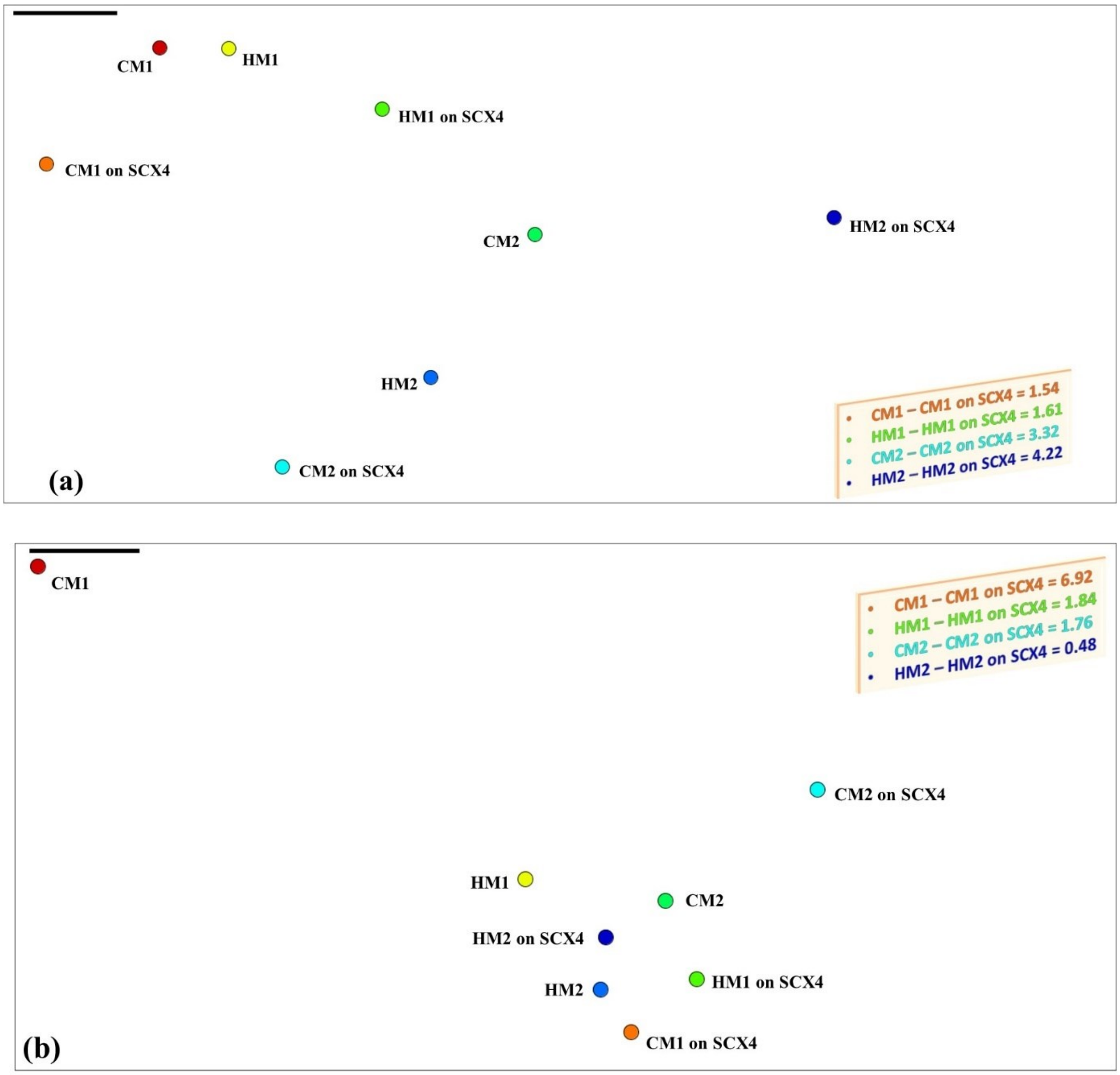

Fig. S10. IDMAP plots for the PM-IRRAS spectra in Fig. 7 in the main text in the range of (a) 3000-2800 and (b) 1800-900 cm^{-1}. The black bar is a reference for measuring relative distances between the data points while disregarding scales on the axes.

References

[1] A.C. Alves, D. Ribeiro, C. Nunes, S. Reis, Biophysics in cancer: The relevance of drug-membrane interaction studies, Biochim Biophys Acta Biomembr 1858 (2016) 2231–2244. https://doi.org/10.1016/j.bbamem.2016.06.025.

[2] W. Szlasa, I. Zendran, A. Zalesińska, M. Tarek, J. Kulbacka, Lipid composition of the cancer cell membrane, J Bioenerg Biomembr 52 (2020) 321–342. https://doi.org/10.1007/s10863-020-09846-4.

[3] T. Zech, C.S. Ejsing, K. Gaus, B. De Wet, A. Shevchenko, K. Simons, T. Harder, Accumulation of raft lipids in T-cell plasma membrane domains engaged in TCR signalling, EMBO Journal 28 (2009) 466–476. https://doi.org/10.1038/emboj.2009.6.

[4] P.L. Yeagle, The membranes of cells, 3rd ed., Academic Press, London, 2016.

[5] K. Pinkwart, F. Schneider, M. Lukoseviciute, T. Sauka-Spengler, E. Lyman, C. Eggeling, E. Sezgin, Nanoscale dynamics of cholesterol in the cell membrane, Journal of Biological Chemistry 294 (2019) 12599–12609. https://doi.org/10.1074/jbc.RA119.009683.

[6] P.J. Rousseeuw, Silhouettes: a graphical aid to the interpretation and validation of cluster analysis, J Comput Appl Math 20 (1987) 53–65. https://doi.org/https://doi.org/10.1016/0377-0427(87)90125-7.
